# A systematic literature review of cybersecurity scales assessing information security awareness

**DOI:** 10.1016/j.heliyon.2023.e14234

**Published:** 2023-03-05

**Authors:** Rohani Rohan, Debajyoti Pal, Jari Hautamäki, Suree Funilkul, Wichian Chutimaskul, Himanshu Thapliyal

**Affiliations:** a School of Information Technology, King Mongkut's University of Technology Thonburi, Bangkok 10140, Thailand; b Innovative Cognitive Computing Research Center (IC2), King Mongkut's University of Technology Thonburi, Bangkok 10140, Thailand; c School of Technology, JAMK University of Applied Sciences, Jyväskylä, Finland; d Department of Electrical Engineering and Computer Science, University of Tennessee, Knoxville, USA

**Keywords:** Cybersecurity, Factor analysis, Information security awareness, Scale development, Reliability, Validity

## Abstract

Information Security Awareness (ISA) is a significant concept that got considerable attention recently and can assist in minimizing the risks associated with information security breaches. Several measurement scales have been developed in this regard, as measuring users’ ISA is paramount. Although ISA specific scales are very important, yet what methodological rigor they use in terms of initial conceptualization of ISA, data collection and analysis during the development, and scale validation of such scales are some unknown aspects. Therefore, we provide a comprehensive review of the existing ISA specific scales to address all the above concerns. A popular method, PRISMA, is utilized, and a total of 24 articles that match with criteria of this research are included for the final in-depth analysis. Also, a holistic evaluation framework is developed containing three phases and 19 criteria. Findings revealed that most studies treat ISA as a multi-dimensional construct, and ISA researchers rarely conduct both pilot testing and pre-text evaluation while validating and refining the initial scales. Additionally, several articles did not report some of the essential elements used for checking the rigor of factor analysis, and evidence for validities of the identified scales is inadequate. Consequently, existing ISA specific scales must be improved both in terms of the methodological thoroughness of the scale development procedure and their validities. Moreover, not only justifying why the development of a new scale is necessary, but also improving the quality of the existing scales by doing multiple iterations is significant in the future. Likewise, the inclusion of all the dimensions of ISA, while generating the initial items pool is an important aspect to be considered. A thorough discussion, recommendations for future research, conclusions, and study limitations are provided.

## 1 Introduction

The recent advancement in digital technologies, interconnectivity, and devices have brought several benefits to organizations in terms of the growing speed of communication, decreasing operating costs, improving system accessibility, and its effect on efficiency and productivity. Nevertheless, organizations, while undergoing digital transformation, encounter the risks of cyber-attacks on their assets [1]. For instance, studies reported that there are more than 4000 ransomware attacks on organizations every day [2,3]. Similarly, another research in Ref. [4] has indicated that more than 330,000 malware incidents globally occur daily. These attacks include phishing scams, malware, ransomware, malicious scans, and various types of social engineering attacks. The consequences and cost of these attacks on organizations are considerable. For example, in 2015, the cost of cyber-attacks was 3 trillion US Dollars worldwide, which increased to 5 trillion in 2017 [5,6], and 6 trillion in 2021 [7,8].

Cybercriminals have targeted various types of private and public organizations like healthcare, education, finance, and other industries. Research states that organizations hold valuable and sensitive information regarding their staff, users, and stakeholders in general [9]. Thus, cybercriminals are interested in targeting this kind of information, which will further assist in repercussions such as the loss of intellectual property, reputation, identity theft, finance, and unauthorized access to computing resources [10,11]. Further, information systems and devices are being increasingly connected to the internet and typically operated in the cloud, due to which cybersecurity has evolved from traditional information security that has resulted in increased risks and a greater demand for security [12]. Therefore, organizations are investing a lot in terms of technology, and they normally go for technical solutions like using firewalls, intrusion detection systems, security algorithms, and a variety of other sophisticated information security tools. Despite these investments, cybercrime continues to be a problem, with substantial data breaches occurring every single day.

One reason behind such data breaches is because human factors have a direct impact on every aspect of information security in organizations, and people are the weakest link in safeguarding the information security systems [13,14]. In this regard, researchers found that 95% of security breaches are caused by human mistakes, implying that technology measures alone cannot ensure a secure environment for an organization's digital assets [15]. For instance, many internet users are still unaware of how their systems can be compromised due to their naive behaviors. Consequently, these users continue to access suspicious websites, create a weak password or share it with others, open links in emails from unknown senders, and expose sensitive information due to other social engineering attacks [16]. This leads to the idea of Information Security Awareness (ISA) [17]. In order to minimize the risks associated with information security breaches, ISA is the best option and plays a significant role in this regard.

Researchers have defined ISA in many ways. For example, ISA has been defined as a method “to educate internet users to be sensitive to the various cyber threats and the vulnerability of computers and data to these threats” [11]. Similarly, it has been defined as “ the degree of users' understanding about the importance of information security, and their responsibilities to exercise sufficient levels of information control to protect the organization's data and networks” [18]. According to these conceptualizations, ISA has two significant aspects. The first aspect stresses how well users in organizations comprehend the significance of information security issues and threats (Knowledge & Awareness), while the second focuses on how good the users follow the organizations' privacy and security rules while using the internet (Activities & Compliance). Hence, it becomes evident that human factors play an important role in the ISA aspect of any organization. Similar findings are reported by previous studies in Refs. [13,14,19,20] show that human awareness is one of the most significant aspects of information security research. Thus, organizations must measure and assess ISA to receive feedback on their users' security behavior and perceptions towards the significance of security, so that they can discover areas of strength and weakness and can further use this information to customize guidelines and awareness programs to enhance the security level of their users.

From the above discussion, it becomes evident that measuring users’ ISA is paramount. One way of doing this is to use comprehensive, validated, reliable, and relevant measurement scales to evaluate ISA. Measurement scales are defined as “*useful tools to attribute scores in some numerical dimension to phenomena that cannot be measured directly*” [21]. With respect to this measurement aspect of ISA, there are few literature reviews that have focused on the methodological aspects of measurement, the different security awareness types, together with their effectiveness [18,22,23,24]. Nevertheless, the current works are limited and not holistic enough. For example, researchers in Ref. [18] investigated the current methods implemented for assessing cybersecurity awareness, the target population, and scope of the existing measures of cybersecurity awareness. Authors in Ref. [22] investigated how ISA is measured and how its measurement can be automated. It mainly addressed security awareness approaches, key challenges, and their solutions. Another systematic review in Ref. [23] was carried out to identify major challenges for the successful implementation of ISA and focused on the factors that have an effect on increasing the effectiveness of the different ISA assessment methods. Similarly, researchers in Ref. [24] concentrated on reviewing the significance and effectiveness of measurement tools for the different ISA programs to check for their adequacy for acquiring the targeted objectives and further identified some measurement scales to check their reliability.

Although these literature review studies addressed issues related to the measurement of ISA, but they fall short in many aspects that are important for the cybersecurity research community. First, since ISA is a broad and multi-dimensional concept, it is not clear as to what are the different dimensions and sub-dimensions that are relevant for its measurement and have been undertaken by current research. Likewise, how much rigor and standardized procedures the current scales follow while measuring ISA is not known. Moreover, scale validation is one critical aspect in the information security domain. For instance, inadequately validated measurement scales may lead organizations to incorrectly assess users’ ISA, and decisions based on those may have devastating outcomes. However, there is limited information related to the extent of the statistical validation the current ISA scales have undergone during their creation. Therefore, it becomes evident that although ISA specific scales are very important, yet what methodological rigor they use in terms of initial conceptualization of ISA, data collection, and analysis during the development of such scales is a grey area. The current research tries to address all these concerns, which is the novelty of this review. More objectively, we try to answer the following four research questions.

RQ1 What is the current state of the ISA specific scales?

RQ2 What are the dimensions and sub-dimensions of ISA that researchers have considered while developing their scales?

RQ3 What is the methodological thoroughness/rigor of the scale development procedure of the measurement scales?

RQ4 What are the reported reliability and validity measures of the identified scales?

This research is separated into seven sections. A detailed methodology of the Systematic Literature Review (SLR) and a quality assessment is presented in Section 2. In Section 3, a holistic evaluation framework is proposed for evaluating the rigor of the scale development process pertaining to ISA, including the reliability and validity measures. Section 4 provides results, where all the four research questions are answered. The discussion is presented in Section 5, together with observations and recommendations for future research. Section 6 provides conclusions. Finally, the study limitations are presented in Section 7.

## 2 Methodology

In order to provide a comprehensive review of the measurement scales used to assess ISA, we base the current study on the PRISMA (“Preferred Reporting Items for Systematic Reviews and Meta-Analyses”) standard [25]. Additionally, the Critical Appraisals Skills Program (CASP) tool [26] is adopted for the purpose of quality assessment of the selected articles while creating the final reading corpus. These are described in detail in the following sub-sections.

### 2.1 PRISMA

**Fig. 1:**
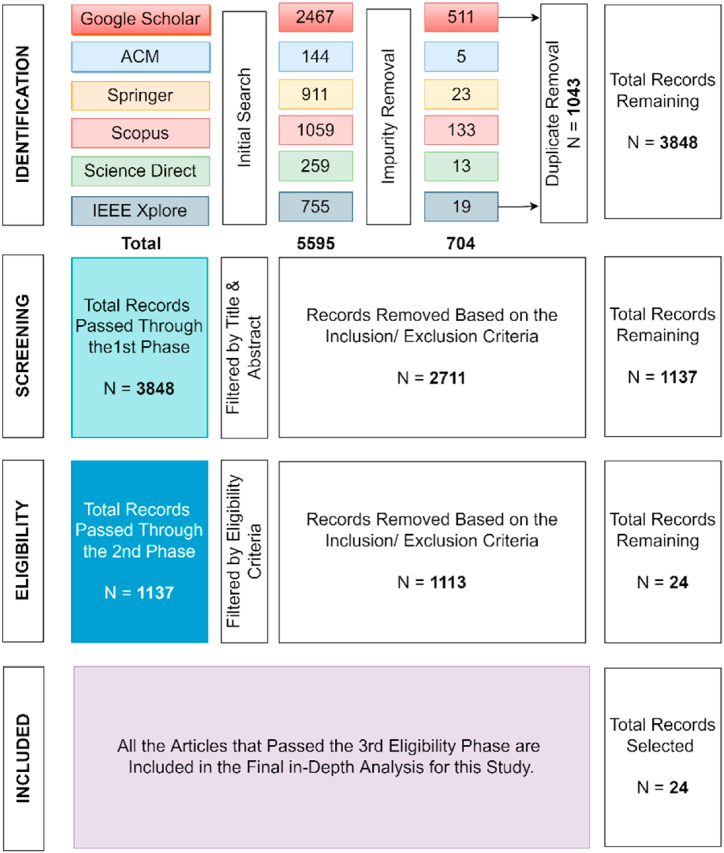
A flow diagram indicating a step-by-step process of identifying and selecting the articles.

PRISMA is the most commonly used, suitable, standardized, and comprehensive method for conducting the SLR [25,27]. In Table 1, we present the advantages and disadvantages of PRISMA, together with our motivation to use this particular method. Although there are some other method/s used for conducting SLR, (e.g., a method used in Ref. [28]), but that is not as standardized, systematic, and widely accepted among the researchers compared to PRISMA [29]. Hence, we initiate the review process by using the PRISMA, which consists of four phases: identification, screening, eligibility, and included. The flow diagram with all the detailed information/statistics is depicted in Fig. 1. Below we explain the four phases in detail.

**Table 1:** Advantages, disadvantages, and motivation of PRISMA [13,25,27].

Advantages	Disadvantages	Motivation
Focuses on specific research objectives	PRISMA does not have quality control (it does not give us any specific methodology to judge the quality of the articles).	To handle the quality control issue, we consider doing an in-depth quality assessment check on the final articles based on the CASP tool (Table 2).
Has an apriori review protocol		
The search strategy is transparent and explicit.		
Standard and well-accepted tool for conducting SLR

#### 2.1.1 Identification phase

##### 2.1.1.1 Selecting databases

We begin the literature review process by identifying a set of scientific and standard databases to get extensive and broad coverage of the relevant literatures. Therefore, we chose Google Scholar, ACM Digital Library, Springer, Scopus, Science Direct, and IEEE Xplore to be used as the primary sources.

##### 2.1.1.2 Selecting keywords

Keywords used for this research are (“information security awareness” OR “cybersecurity awareness”) AND (scale OR questionnaire OR measure* OR assess* OR evaluat*). Keeping in mind the research objectives, the keyword combinations (search query) are developed in such a way to capture both generic as well as specific research items. For instance, keywords (“information security awareness” OR “cybersecurity awareness”) are general ones and combined with the specific keywords (scale OR questionnaire OR measure* OR assess* OR evaluat*).

##### 2.1.1.3 Initial search

The search query we used to retrieve the related literatures resulted in a total of 5595 records. Later on, we used two filtering criteria (impurity removal and duplicate removal) to restrict the search based on our objectives. The review was limited to articles published between the years (2010–2022), and the search was done in April 2022 with a repetition in July 2022 to check for any additional literatures.

##### 2.1.1.4 Impurity removal (Inclusion/exclusion criteria)

The search concentrated on articles published in English, and only full-text, conference, and peer-reviewed journal articles. We considered these criteria because journal and conference articles normally undergo a thorough peer review process, due to which they report more innovative and accurate research findings compared to other sources. Also, full-text articles indicate the availability of full and comprehensive evaluation processes. Therefore, news articles, non-technical magazines, short abstracts, book chapters, and annual reports talking about information security or cybersecurity measurements/awareness were all excluded. As a result, a total of 704 records were eliminated int this initial phase.

##### 2.1.1.5 Duplicate removal

In this step, we eliminated all the duplicate records, which summed up to 1043. As we included (Google Scholar and Scopus) in our search, it resulted in many duplicate records. Finally, the total remaining articles after the identification phase were 3848.

#### 2.1.2 Screening phase

After the identification phase, the title and abstract of each of the selected articles were read in the screening phase, and a total of 1137 articles were included to be carried over to the next (eligibility) phase. This screening was done based on a fresh set of inclusion/exclusion criteria designed specifically for this phase, as shown below.

##### 2.1.2.1 Inclusion and Exclusion Criteria

Articles that mentioned information security awareness or cybersecurity awareness together with words like (measurement, assessment, evaluation, scale, or questionnaire) either in the title, abstract, or as keywords were included. This process eliminated 2711 records that only mentioned information security awareness, and/or cybersecurity awareness, or other security issues in general, but did not provide a mechanism to assess the scenario.

#### 2.1.3 Eligibility phase

A total of 1137 articles that passed through the second screening phase were selected for full text reading in the eligibility phase. All the related articles were accessed using the library service provided by the first author's university. The filtering criterion used to assess the articles in this phase is provided below.

##### 2.1.3.1 Inclusion and Exclusion Criteria

*First*, those articles were excluded that used existing scales, instead of developing a new one. A total of 577 articles were eliminated based on this criterion. For example, authors in Ref. [30] examined the ISA of bank employees and used an existing scale called the Human Aspects of Information Security Questionnaire (HAIS-Q) instead of developing their own scale, due to which it was excluded. Similarly, for some of the articles, although after reading the title and abstract, it seemed that they developed their own scale, however after reading the full text, it was found that they used some of the existing scales. For instance, the authors in Ref. [31] tried to investigate whether factors like internet addiction and attitude of internet users towards cybersecurity predict the awareness and engagement in risky behaviors. They used an already developed scale called Risky Cybersecurity Behaviors Scale for measuring awareness. *Second*, those articles irrelevant to our topic (discussing about general security management and measurement issues instead of ISA) were excluded (n = 489). For example, authors in Ref. [17] investigated information security culture and how information privacy can be incorporated. Similarly, the authors in Ref. [32] evaluated the cybersecurity judgment behavior of learners to determine if there are any particular weak links. All such articles that did not deal with ISA directly were removed in this full-text reading phase. *Third*, all the literature review articles, such as [22,23,24], were also excluded (n = 45). Fourth, we included research articles that focused on quantitative or mixed-method techniques for developing or validating the scales. Hence, articles focusing only on qualitative techniques like interviews or theoretical approaches without empirical research were excluded (n = 2). For instance, authors in Ref. [33] developed scales for assessing the relevance of internet users' behaviors. They utilized only a qualitative approach with discussion for validating their scale, due to which it was removed.

Finally, 24 articles matched with our context (Fig. 1). One thing worth mentioning over here is that the number of articles alarmingly decreased in the eligibility phase. For example, roughly only 2% of the articles passed the final eligibility criterion when compared to the previous screening phases. There may be two possible reasons for this. First, majority of the articles discussed different scales of ISA; however, they did not develop any scale of their own. Rather they used some form of already developed scale. Second, there were several articles that used theoretical models to explain the attitude and users' intention toward ISA. However, neither they developed any new scales nor utilized any current measures for describing any particular phenomena.

#### 2.1.4 Included phase

All the articles that passed the third eligibility phase were included in the final in-depth analysis for this study. As a result, we have included 24 articles that matched with all the criteria of this research. During all the phases of the PRISMA standard, five researchers were involved in assessing the articles in an independent way. In case of any disagreements regarding the suitability or eligibility of any specific article, we tried to resolve them through a mutual discussion. Still, if there was a difference in opinion, the article was included. As mentioned previously, since roughly only 2% of the articles passed from the initial screening to the final eligibility and included phase, we decided to do an in-depth quality assessment check on the final article corpus to judge their suitability.

### 2.2 Quality assessment

The final corpus containing 24 articles was further evaluated using a quality assessment criterion based on the CASP tool [26]. This assessment was performed to make sure that the contents of all the selected articles were matching with our research objectives. Articles that passed these criteria demonstrated adequate validity to be included for the full-text in-depth analysis. For the evaluation purpose, we weighted our assessment by applying a 3-point scale (0, 1, and 2) to each criterion, where 0 means that the criterion is not met at all, 1 for criterion partially met, and 2 for criterion totally met. The quality assessment items, along with the results, are presented in Table 2. The number of articles that passed a particular criterion is written under either of the three columns. For instance, considering criterion #1 (the aim of research), 22 articles totally passed this criterion, and two studies [34,35], partially passed. Strangely, there is only one article [36] that considered the ethical issues (criterion #7). However, as these articles passed all the other 9 criteria, therefore, we included all of them for full-text analysis. Overall, none of the articles were rejected in this quality assessment phase .

**Table 2:** Quality Assessment According to the CASP tool [26].

No	Quality Assessment Criteria	Totally Met	Partially Met	Not Met
1	Is there a clear statement about the aims of the research?	22	2	0
2	Is the methodology appropriate?	17	7	0
3	Is the research design appropriate to address the aims of the research?	16	8	0
4	Is the recruitment strategy of the participants appropriate and well-explained to the aims of the research?	18	6	0
5	Is the data collected in a way that addressed the research issue?	21	3	0
6	Has the relationship between researcher and participants been adequately considered?	18	6	0
7	Have ethical issues been taken into consideration?	1	0	23
8	Is the data analysis sufficiently rigorous?	14	10	0
9	Is there a clear statement of findings?	20	4	0
10	How valuable is the research?	20	4	0

## 3 Evaluation framework

For answering the research questions, especially (RQ_3_ and RQ_4_), specific criteria must be formulated for evaluating the rigor of the scale development procedure together with the reliability and validity of the measurement scales. For achieving this, we followed the guidelines and the set of best practices outlined by some of the popular and widely accepted research related to scale development. For example, authors in Ref. [21] identified the current limitations in the scale development process and proposed recommendations for future research. Another study in Ref. [37] provided a guide for researchers regarding the ten steps that must be followed during the scale development process. Likewise, researchers in Ref. [38] proposed the best practices for developing and validating new scales, and authors in Ref. [39] provided guidelines for developing a better measurement scale. Finally, a comprehensive review on scale development was conducted by authors in Ref. [40] that not only provided the set of best practices, but also proposed recommendations for improving the overall scale development process.

**Fig. 2:**
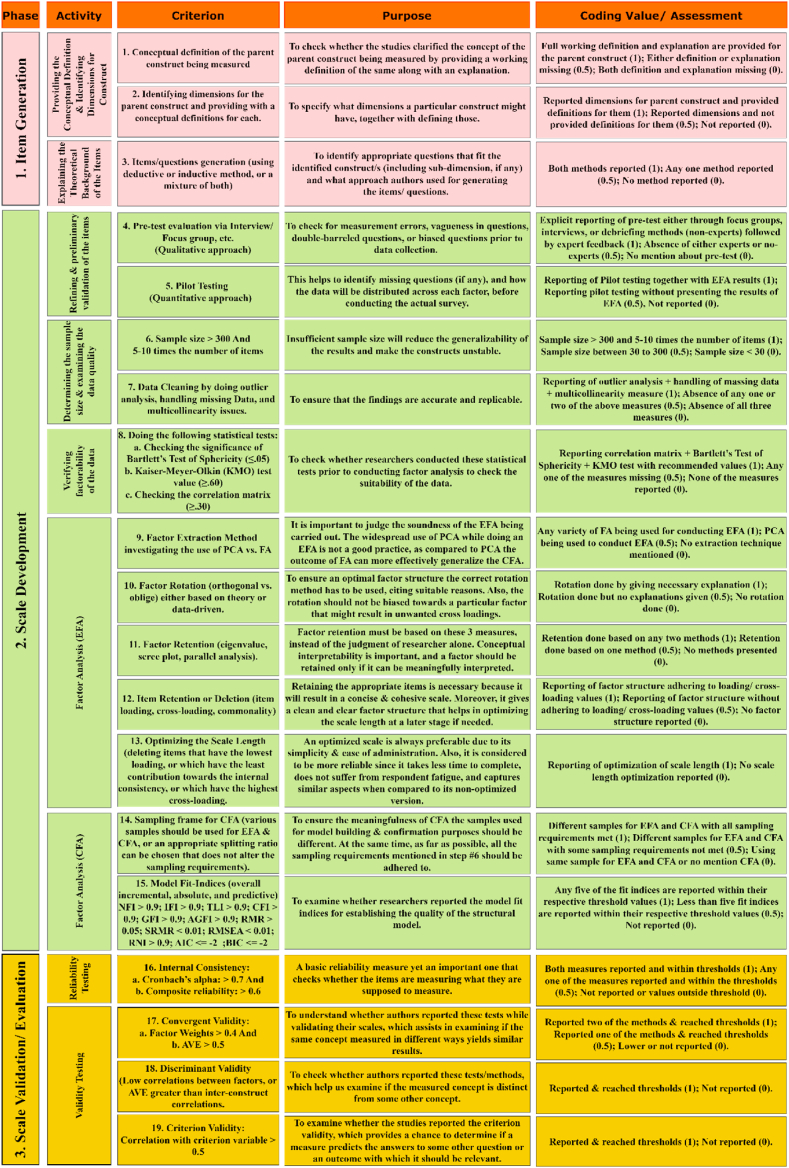
Framework for evaluating the rigor, reliability, and validity of the measurement scales.

We developed our own evaluation framework by incorporating the core concepts presented by the above-mentioned studies. The proposed evaluation framework is presented in Fig. 2. The entire framework is separated into three main phases: item generation, scale development, and scale evaluation/validation. Each phase has multiple activities, and each activity is evaluated by specific criteria/criterion. The activities are evaluated based on three levels of score that are coded as fulfilled (score of 1), partially fulfilled (score of 0.5), and not reported (score of 0).

We would like to clarify that we do not give scores or evaluate the studies as to how good or bad these articles are. The scoring system assigned for every criterion is only for evaluation purposes based on the best practices drafted by some widely accepted research associated to scale development. Therefore, instead of judging the quality of the articles, we wanted to highlight how rigorously they follow the process of developing a new scale as it is very important for critical use cases like cybersecurity. The analysis of the first two phases of the evaluation framework (item generation and scale development with their set of criteria) will help us in answering RQ_3_, while the third phase (scale evaluation/validation with its set of criteria) will assist us in answering RQ_4_. We describe all the three phases in detail below.

### 3.1 First phase (Item generation)

The first phase of the evaluation framework is item generation, which focuses on the conceptual definition of the construct, identifying possible dimensions of the construct, and selecting the appropriate questions/items on a per construct basis. All the relevant criteria are explained below.

#### 3.1.1 Criterion 1 (Conceptual definition)

The first thing to start with the scale development process is to clarify the conceptual meaning by providing a working definition of the construct being measured (ISA for the present case). A construct *“refers to the concept, attribute, or unobserved behavior which is the target of a study”* [21]. Any ambiguous or unclear definition of the construct will be misleading. In contrast, a well-defined construct imparts insight into the phenomenon being measured, specifies its scope, and makes the process of item/question generation easy. For example, assuming that ISA is considered as a construct, it should be defined by researchers at the very first stage of the scale development procedure. Therefore, in our proposed evaluation framework, we check and report whether researchers defined the constructs in their studies and accordingly proposed the evaluation scheme. We give a full score of 1 to those articles that provided a full working definition with a proper explanation for the parent construct, a partial score of 0.5 for either definition or explanation being missed for the parent construct, and a score of 0 where both definition and explanation are not provided.

#### 3.1.2 Criterion 2 (Identifying the dimensions)

After the conceptual definition of the parent construct, the second criterion that needs to be assessed is the possible dimensions of the construct. This contributes to a more solid understanding of the construct and the relevant items that describe each dimension. Additionally, identification of possible sub-dimensions for each dimension is also necessary to comprehend what exactly each dimension focuses on and further help us understand what we measure in a particular dimension. Therefore, in our evaluation framework, the criterion was considered as fulfilled if the articles reported dimensions for the parent construct and provided a definition for them (give a score 1), partially fulfilled if dimensions were reported but not defined (give a score 0.5), and not fulfilled if nothing was reported (give a score 0).

#### 3.1.3 Criterion 3 (Item generation)

The last criterion of the first phase is to generate the questionnaire/items. Items can be identified or developed via three possible approaches: deductive and inductive, or a mixture of these two. The deductive approach is based on the description of the related constructs together with the dimension(s) and sub-dimension(s), followed by the identification of the items [38]. Normally, this procedure is carried out through a holistic literature review of the existing scales. The second inductive approach is based on individuals' opinions or feedback that can be done through various qualitative techniques [39]. For example, using interviews and focus groups for obtaining the experts’ opinions regarding the items and the identification of relevant dimensions/sub-dimensions. It is highly recommended that the initial pool of items should be simple, distinct, specific, and reflect their original purpose [40,41]. Accordingly, we created the evaluation framework giving a full score of 1 to those studies that reported the use of both the methods, a partial score of 0.5 to those which reported the use of either one of the techniques, and a score of 0 if no method is reported.

### 3.2 Second phase (Scale development)

The second phase of the evaluation framework is the scale development, which includes refining the scale, determining the appropriate sample, examining the data quality, verifying the factorability of the data, and finally, conducting a factor analysis. All these activities and their respective criteria are discussed below in detail. One important activity after proposing the initial scale is to refine it and check for the initial validity. This should be done by two ways: a pre-test evaluation (qualitative technique), followed by a pilot testing (quantitative technique). We would like to emphasize that although pre and post-tests have similar objectives of further scale refinement, we included both these in our evaluation framework (criteria 4 and 5) because while the former addresses issues related to ambiguity, confusion, difficulty, or missing questions, the later provides a rehearsal of the actual survey that is going to be conducted under actual field conditions that enables to identify how the data will be distributed among the different constructs.

#### 3.2.1 Criterion 4 (Pre-test evaluation)

Pre-tests are carried out before the launch of the main survey. It carefully considers assessing the entire questionnaire, including the conceptualization of the dimensions and sub-dimensions, questionnaire wording, vagueness of questions, their representativeness, and the extent to which the questions reflect their intended meaning. This assessment should be done both by experts and the target population for whom the scale is being developed. Experts are well-educated in a particular domain and can give their opinion both from a methodological perspective and the domain knowledge. On the other hand, the target population consists of the potential users of the scale. Ideally, pre-tests should cover both these groups and can be done using qualitative techniques like interviews, focus groups, group or individual debriefings, etc. [21,42]. For the scoring system, we gave a full score of 1 for those works that conducted pre-tests by considering both experts and the target population, gave a partial score of 0.5 for those works that involved only one of the groups, and a score of 0 for those where nothing was mentioned about this criterion.

Criterion 5 (Pilot Testing): It is a quantitative method that is conducted before the actual survey, and it plays a significant role in determining how data will fall around each factor and specifying the missing questions. Therefore, Exploratory Factor Analysis (EFA) is highly recommended for the data collected during the pilot testing phase, and the sample size should be between (50–100) participants [37,40]. While creating the evaluation framework, we decided to give the full score of 1 to those works that reported pilot testing and presented the EFA results also, a score of 0.5 to those that only mentioned about pilot testing without presenting the EFA results, and a score of 0 that did not mention anything.

#### 3.2.2 Criterion 6 (Determining the sample size)

The sixth criterion that we included in our evaluation framework was to check whether scholars adhered to the rules in terms of the minimum sample size. Before conducting the actual survey, it is necessary to decide on a suitable sample size. A small sample brings inconsistency to the factors, reduces generalizability, and consequently produces biased measurement results [37,42]. Hence, an optimal sample size is preferable, which is good not only in terms of statistically significant results but also helps in getting higher factor loadings and obtaining more stable scales. Although there is no uniform consensus among researchers regarding what the optimal sample size should be, the most recommended ones in literatures suggest a minimum sample size of 300 and 5–10 times the number of items (5:1 or 10:1) [21,38]. For evaluation purposes, we gave a full score of 1 to those works that maintained the minimum threshold sample size as recommended above, gave a score of 0.5 to those works that did not maintain the threshold, and a score of 0 was given to those works that reported a sample size of less than 30. We chose 30 as the cut off value since it is the minimum requirement for the Central Limit Theorem to hold true.

#### 3.2.3 Criterion 7 (Data quality)

This is another important criterion that refers to the data cleaning process undertaken after its collection (e.g., checking for missing data, outliers, and multicollinearity issues). Researchers must report how missing data was handled and carry out an outlier analysis together with reporting the precautionary steps undertaken to prevent multicollinearity issues during data collection. Additionally, statistical measures like Variance Inflation Factor (VIF) values must be reported [21,43]. A full score of 1 is given if all the three measures of outlier analysis, missing data, and multicollinearity are reported, whereas if any one or two of the measures are missing, a score of 0.5 is given. In case none of the measures are reported, we give a score of 0.

#### 3.2.4 Criterion 8 (Verifying factorability of the data)

The eighth criterion of the scale development procedure is to investigate whether the collected data is suitable for factor analysis. It is essential to examine three aspects: correlation matrix, Kaiser-Meyer-Olkin (KMO) test, and Bartlett's test of sphericity. The recommended criteria/value for KMO is ≥ 0.60, Bartlett's chi-square should be significant at ≤ 0.05, and the correlation matrix should have items having a value of ≥0.30 [37,44]. Current literatures recommend all the three tests, but two of them, particularly KMO and Bartlett's test, are mandatory [21,45]. Therefore, while giving the scores, we gave a full score of 1 to those articles that reported all the three measures with the recommended values, a score of 0.5 if any one of the measures is missing, and a score of 0 if none of the measures are reported. In addition, one of the main activities of the evaluation framework is to apply factor analysis to the collected data. Ideally, an EFA should be followed by a CFA. Moreover, different datasets should be used for conducting the EFA and CFA. Therefore, we treat these two as different and distinct activities. The rigor of EFA is evaluated by checking five important aspects: factor extraction method, factor rotation, factor retention, item deletion or retention, and optimizing the scale length.

#### 3.2.5 Criterion 9 (Factor extraction)

Depending on different statistical theories, there are various factor extraction methods. However, Principal Component Analysis (PCA) and Common Factor Analysis (FA) are the most popular ones. Although it is an ongoing and unresolved issue regarding the preferential use of PCA over FA (e.g., maximum-likelihood or principal-axis factoring methods), current research has shown that PCA is inherently different from FA both conceptually and mathematically [46]. While PCA is more suitable for dimension reduction problems, the purpose of FA is to understand the latent constructs that account for the shared variance among the different items. Therefore, choosing an appropriate FA technique over PCA should always be the preferable factor extraction method. Consequently, while designing our evaluation framework, a full score of 1 is given if any form of FA technique is applied for the EFA phase, while if PCA is employed a score of 0.5 is awarded. If the name of the method used for factor extraction is not mentioned, we give a score of 0.

#### 3.2.6 Criterion 10 (Factor rotation)

This is another essential aspect to clearly identify the different dimensions. There are two kinds of rotation available (orthogonal and oblique). The type of rotation method to be used during the initial FA is based either on theory or the collected data. Whatever extraction method is chosen, researchers must provide with adequate rationale for selecting either an orthogonal or oblique rotation method. Moreover, the rotation method should not be biased against finding a general factor and create more cross-loadings in the procedure that might be a problem. For uncorrelated constructs, orthogonal rotation like Varimax is preferable, whereas, for correlated constructs oblique rotation methods like Promax create a better factor structure [47]. Nevertheless, whatever rotation method is chosen, a proper reasoning must be provided. Accordingly, in our evaluation framework, we give a score of 1 if the authors mention that rotation was conducted by giving proper reasoning, a score of 0.5 if no rationale is provided for the factor rotation, and a score of 0 if there is no mention of factor rotation without any reason.

#### 3.2.7 Criterion 11 (Factor retention)

Different criteria may be used for retaining the ideal number of factors based on the item loadings. Some of the most commonly used ones are eigenvalue greater than 1 rule, Scree plot, and Parallel analysis [37,38]. A higher eigenvalue is indicative of a greater proportion of variance, and any value less than 1 indicates potentially unusable factors [48,49]. Scree plot provides with a visual inspection of such eigenvalues that are arranged in a descending order. In parallel analysis, the optimal number of factors is determined by comparing the eigenvalues in the original dataset with a randomly ordered dataset [47]. Using any two of these methods are recommended by the literature; otherwise, at least one of them is mandatory [44]. Accordingly, while creating the evaluation framework, we give a score of 1 if any two factor retention methods are provided, give a score of 0.5 if only one factor retention method is presented, and give a score of 0 if no retention methods are mentioned.

#### 3.2.8 Criterion 12 (Item retention and deletion)

Retaining and deleting items that load onto one (or multiple) factors is also an important issue to consider. With regards to what should be the minimum loading value of each item, there are several recommendations; however, 0.40 is often considered to be the bare minimum [38]. Likewise, if an item cross-loads onto multiple factors, such cross-loadings should have less than 0.15 difference from an item's highest factor loading. Moreover, items should also be deleted if they load with the same minimum threshold values (or greater) across multiple factors [21]. For ensuring the reproducibility of research and acknowledging the fact that scale development is often an iterative process, publishing the final factor structure becomes extremely important as it offers various insights. Consequently, we incorporated this aspect into our evaluation framework by giving a full score of 1 if the factor structure is published and the loading/cross-loading criteria are adhered to, a score of 0.5 if the factor structure is published but any of the criteria is not satisfied, and a score of 0 if the final factor structure is not published.

#### 3.2.9 Criterion 13 (Optimizing the scale length)

As a last step of the EFA, in order to ensure a good quality scale, it is necessary to assess the trade-off between the length of the scale and its reliability. Although longer scales are typically more reliable, it might be problematic to actually administer these types of scales commercially due to lack of respondent motivation, time, and fatigue. Therefore, it is a reasonable idea to go for a trade-off by sacrificing a small degree of internal consistency for shortening the scale. For example, if a factor has more than the desired number of items, then the researcher can delete the item that has the minimum loading or the item that has the least contribution to the internal consistency of the scale. However, such optimizations should not degrade the quality of the factor structure, item communalities, or cross-loadings. While designing the evaluation framework, we give the full score of 1 if scale optimization has been reported, else we give a score of 0.

Once the factor structure is established through EFA that represents the measurement model, a CFA should typically be conducted to confirm the hypothesized model. The rigor of CFA is assessed from two aspects: sampling frame, and model-fit indices. The importance and motivation behind including both these aspects in the evaluation framework are explained below.

#### 3.2.10 Criterion 14 (Sampling frame)

This is one very important aspect that dictates the quality of CFA. The same dataset should not be used for carrying out CFA that was used for conducting the EFA. Depending upon the initial sample size or the number of items, different split ratios can be used that do not affect the quality of the data analysis. Else, it is always recommended to carry out the CFA on a fresh sampling frame [49,50]. With regards to the pre-requisites of the sampling frame, all the requirements that we mentioned previously for selecting a suitable sample during the EFA phase still hold true for CFA. Accordingly, while creating the evaluation framework, we gave full a score of 1 if the sampling frame is different for EFA and CFA with all previous sampling requirements being met, a score of 0.5 if the sampling frame is different for EFA and CFA, but the sampling requirements are not met, and a score 0 if EFA and CFA are carried on the same sample or if conducting of CFA is not mentioned altogether.

#### 3.2.11 Criterion 15 (Model fit indices)

For CFA, it is customary to report the overall model fit as well as other types of fit indices. Typically, the overall model fit is represented by the chi-square test statistic and the associated degrees of freedom [51]. Additionally, incremental, absolute, and predictive fit indices may also be reported [51,52]. Normed Fit Index (NFI), Incremental Fit Index (IFI), Tucker-Lewis Index (TLI), Comparative Fit Index (CFI), and Relative Non-centrality Index (RNI) are some of the measures that should be reported as a part of incremental fit indices. Absolute fit indices include measures such as Goodness-of-Fit Index (GFI), Adjusted Goodness-of-Fit Index (AGFI), Hoelter *N*, Root Mean Square Residual (RMR), Standardized Root Mean Square Residual (SRMR), and Root Mean-Square Error of Approximation (RMSEA). Finally, Akaike's Information Criterion (AIC), Bayesian Information Criterion (BIC), and Expected Cross-Validation Index (ECVI) may be used as the measures of predictive fit indices. Recommended threshold values for all the indices are shown in Fig. 2.

As evident since a variety of fit indices exist, we simplified our evaluation framework by giving a full score of 1 if any five of the fit indices mentioned above are present, give a score of 0.5 if at least the overall model fit is reported, and a score of 0 if nothing about model fit is mentioned.

### 3.3 Third phase (Scale validation/evaluation)

Scale validation is one of the prominent aspects of scale development process. For instance, inadequately validated measurement scales may lead organizations to incorrectly assess the target construct [39]. Hence, decisions based on this may have devastating outcomes [18,21]. Therefore, it is mandatory to check the validity and reliability of the developed measurement scales, which can be done by the different well-established reliability and validity measures. The first significant criterion for evaluating the quality of a particular measurement scale is to check its reliability. In this regard, researchers recommend internal consistency [21].

#### 3.3.1 Criterion 16 (Reliability testing)

In terms of internal consistency, several statistics have been developed by researchers to estimate reliability, but normally it is evaluated based on Cronbach's alpha [42]. The recommended threshold value for Cronbach's alpha should be at least 0.7 [48]. An alternative approach for checking the internal consistency is the composite reliability (CR) coefficient. The recommended threshold value for CR should be greater than 0.6 [38]. We chose both these measures as indicators of internal consistency because the way these are evaluated are fundamentally different. For instance, Cronbach's alpha considers all the factor loadings to be the same for all the items; however, CR takes into consideration the variable item factor loadings. In our evaluation framework, a full score of 1 is given if both these measures are present, a score of 0.5 is given if at least one is present, and if none is mentioned, a score of 0 is given.

After the reliability, validity is another significant aspect defined as “the extent to which an instrument indeed measures the latent dimension or construct it was developed to evaluate” [38]. It is mainly examined by the construct validity (a mixture of convergent, discriminant, and criterion validity).

#### 3.3.2 Criterion 17 (Convergent validity)

It is the degree to which a construct measured by several methods achieves similar results and is usually calculated based on factor weights (loadings). The recommended threshold value should be greater than 0.4 [39]. An additional approach is the average variance extracted (AVE) coefficient, and the acceptable values should be higher than 0.5. In our proposed evaluation framework, a full score of 1 is given if both these measures are reported, a score of 0.5 is given if at least one is present, and if none is mentioned, a score of 0 is given.

#### 3.3.3 Criterion 18 (Discriminant validity)

This type of validity is defined as “the extent to which a measure is novel and not simply a reflection of some other construct” [53]. This is usually estimated on the basis of the square root of AVE, which should be greater than inter-construct correlations or the correlations between factors [38]. Thus, in our evaluation framework, a full score of 1 is given if results of discriminant validity are present, a score of 0 is given if the results are not presented.

#### 3.3.4 Criterion 19 (Criterion validity)

The third significant validity is the criterion validity, “which describes the extent to which the measure correlates with an expected outcome or a variable with which it is supposed to be highly correlated” [42]. Thus, the recommended threshold value of the correlation should be higher than 0.5. Therefore, in our proposed evaluation framework, a full score of 1 is given if the result of criterion validity is reported, and a score of 0 is given if the result is not reported.

## 4 Results and analysis

In this section, we try to answer the four research questions that had been proposed earlier in order to understand the current state of the existing scales developed for measuring the ISA and further provide foundations for the future development of high-quality cybersecurity measurement scales.

### 4.1 RQ_1_. What is the current state of ISA specific scales?

For answering the first research question, a summary of the basic characteristics, such as the number of items developed by each study, their objectives, and the target population, together with the sample size of all 24 selected articles are presented in Table 3, 3a, 3b. In terms of the items, both the initial items pool and the final proposed items for each study is presented. Similarly, the objectives of all the selected articles are given. In this respect, it is worth mentioning that scale development is a secondary objective for some of the studies, like [54,55,56,57]. Their first objective was to propose some theoretical models and then develop measurement scales for describing their research models.

**Table 3:** General characteristics of all the 24 selected articles.

No	No of Items	Objective	ISA Definition Aspects	Target population
[34]	Initial items pool (n = 33) Proposed items (n = 17)	The study focuses on developing a scale for measuring the users' risky cybersecurity behaviors, awareness, and vulnerabilities.	Knowledge & Awareness	College Managers (Academia) Sample size not reported
[35]	Initial items pool (n = 69) Proposed Items (n = 28)	To develop a scale to measure whether cybersecurity events/training programs impact cybersecurity awareness.	Knowledge & Awareness	University learners (Academia) Main Survey (214)
[36]	Initial items pool (n = 74) Proposed Items (n = 25)	Developed instruments for measuring the users' cybersecurity perceptions and awareness concerning e-mail usage, social engineering, passwords, social media applications, and other online services.	Knowledge & Awareness	University Students (Academia) Main survey (n = 320)
[54]	Initial items pool (n = 45) Proposed items (n = 6) for ISA	Developed a scale for measuring general information security awareness.	Activities & Compliance	Employees working at organizations and accessed the internet. (Industry) Main Survey (n = 928)
[55]	Initial items pool (n = 85) Proposed items (n = 25) for ISA	To develop and validate scale for measuring a model that contains human behavioral factors, with a focus on ISA.	Activities & Compliance + Knowledge & Awareness	Employees from different organizations (Industry) Main survey (n = 1085)
[56]	Initial items pool (n = 87) Proposed items (n = 10) For ISA	The focus is on the human aspects of ISA, so developed instruments to aware users and staff regarding the security policies and procedures in a library context.	Knowledge & Awareness	Professional and staff working in 4 libraries (Academia) Main survey (n = 69)
[57]	Initial items pool (n = 49) Proposed items (n = 10) For ISA	The article focuses on developing measurement instruments for assessing internet users' security awareness and attitude.	Knowledge & Awareness	Employees from different organizations (Industry) Main survey (4296)
[58]	Initial items pool (not reported) Proposed items (n = 21)	The purpose of the research is to develop and validate measurement instruments for assessing the human aspects of ISA.	Knowledge & Awareness + Activities & Compliance	Employees working in different organizations (Industry) Main survey (n = 1073)
[59]	Initial items pool (not reported) Proposed items (n = 63)	The study focuses on developing and validating the human aspects of ISA measurement tools.	Knowledge & Awareness + Activities & Compliance	University students and employees from other organizations (Academia + Industry) Main survey (n = 1112)
[60]	Initial items pool (Not reported) Proposed items (n = 9)	The aim is to investigate the feasibility of an information security vocabulary test and develop a scale to evaluate the ISA levels of users.	Knowledge & Awareness	University students from different departments (Academia) Sample size is not Reported

**Table 3a:** General characteristics of all the 24 selected articles (continued).

No	No of Items	Objective	ISA Definition Aspects	Target population
[61]	Initial items pool (n = 19) Proposed items (n = 11) for ISA	The study concentrates on developing instruments for examining ISA and information security training.	Activities & Compliance	Participants of 200 public & private organizations (Academia + Industry) Main survey (2000+)
[62]	Initial items pool (n = 43) Proposed items (n = 7)	The goal of the study is to develop and validate scales to measure the ISA and users' deviant behavior.	Knowledge & Awareness	University students and employees working in various organizations. (Academia + Industry) Main survey 1 (n = 1000)
[63]	Initial items pool (n = 37) Proposed items (n = 17)	The main purpose of this research is to develop a reliable instrument for measuring users' ISA.	Knowledge & Awareness	University students from various faculties (Academia) Main survey (n = 135)
[64]	Initial items pool (n = 30) Proposed items (n = 16)	The focus is mainly on developing scale that measures security behaviors and awareness of the internet users.	Knowledge & Awareness	Employees from organizations. (Industry) Main Survey (n = 503)
[65]	Initial items pool (n = 89) Proposed items (n = 30)	Developed and tested scales to measure security awareness and internet users' risky behavior.	Knowledge & Awareness	Students and academic's staff (Academia) Main survey (n = 385)
[66]	Initial items pool (n = 48) Proposed items (n = 7) for ISA	The article concentrates on the internet users' security complaint behaviors and ISA levels, thus developing a scale for that.	Knowledge & Awareness + Activities & Compliance	University students (Academia) Main survey (n = 301)
[67]	Initial items pool (n = 24) Proposed Items (n = 7) for ISA	Researchers in the study developed and tested a scale measuring organizational information security awareness.	Knowledge & Awareness + Activities & Compliance	Employees and managers from organizations (Industry) Main Survey (n = 323)
[68]	Initial items pool (n = 71) Proposed Items (n = 18)	Authors in the study investigated the cyber hygiene behavior of users, which is generally measured based on awareness as it always precedes behavior.	Knowledge & Awareness	General internet user from different countries (not mentioned specific group) Main Survey (n = 323)
[69]	Initial items pool (n = 25) Proposed Items (n = 17)	Researchers in the article developed and validated instruments for measuring Mobile ISA.	Knowledge & Awareness	University Students (Academia) Main Survey (n = 562)
[70]	Initial items pool (n = 75) Proposed Items (n = 11)	Authors developed and tested measurement tools for assessing human aspect of ISA and focused on employees' information security behavior.	Knowledge & Awareness	University Students. And administrative staff (Academia) Main survey (n = 263)
[71]	Initial items pool (not reported) Proposed items (n = 12)	The study developed a scale for measuring the users' awareness regarding disclosure of information on social media platforms, unintentional threats, password management, etc.	Knowledge & Awareness	General internet user from different countries (not mentioned specific group) Main Survey (n = 54)

**Table 3b:** General characteristics of all the 24 selected articles (continued).

No	No of Items	Objective	ISA Definition Aspects	Target population
[72]	Initial items pool (n = 90) Proposed Items (n = 34)	Researchers in the article developed and validated a measurement scale for internet users to determine their ISA levels.	Knowledge & Awareness	University Students (Academia) Main survey (n = 442)
[73]	Initial items pool (n = 69) Proposed Items (n = 28)	The purpose was to develop a scale for measuring the awareness and security behavior of the users while using social networking platforms.	Knowledge & Awareness	University students from various departments. Social Media Users. (Academia) Main Survey (n = 585)
[74]	Initial items pool (n = 27) Proposed Items (n = 27)	To develop measurement scale for investigating attitude of college learners toward ISA	Knowledge & Awareness	College Students (Academia) Main survey (n = 196)

For example, the authors in Ref. [55] focus on ISA, security culture, and further try to predict the social engineering and security behavior of the participants. Therefore, they developed a theoretical model investigating employees' intention to resist social engineering attacks. For testing their models, researchers in such studies developed scales for measuring different constructs, including ISA. Another issue is that two of the studies [58,59] focused on the same scale, “Human Aspects of Information Security Questionnaire (HAIS-Q)”. Still, we included both in the final analysis as their objectives were different. For example, authors in Ref. [58] developed 21 focus and sub-focus areas and further investigated the relationship between knowledge, attitude, and behavior. In contrast, researchers in Ref. [59] developed 63 items for seven dimensions based on the HAIS-Q and further validated their scale in two separate studies with different sample populations.

**Fig. 3:**
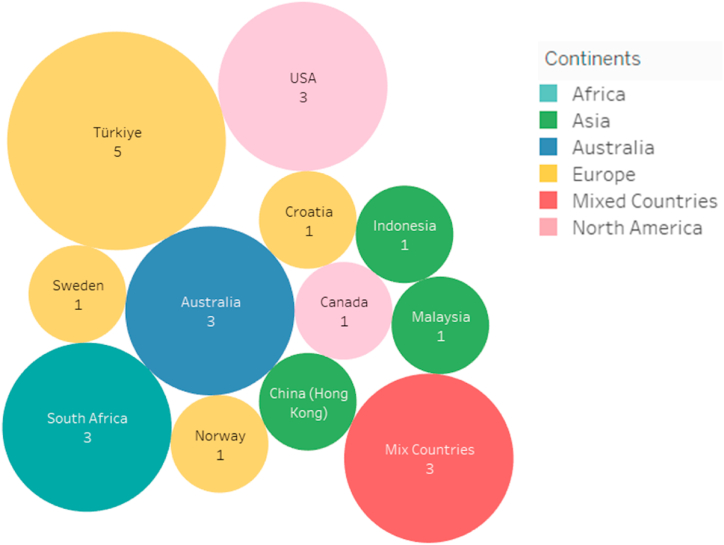
A country-based distribution of the selected articles. The numbers in circles show the number of articles conducted in a particular country/continent.

**Fig. 4:**
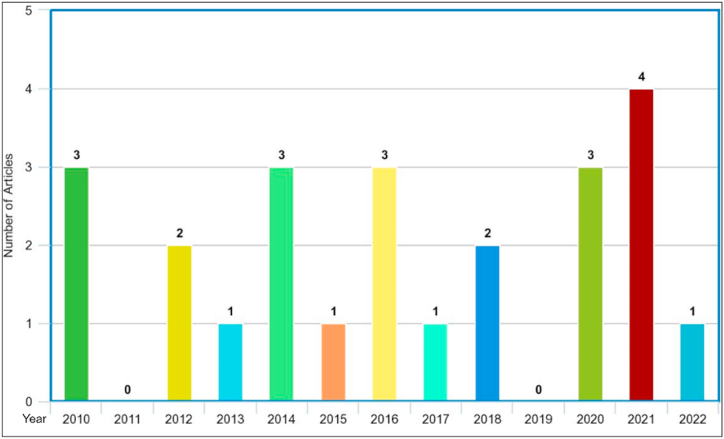
Year-based distribution of the selected articles. The X-axis indicates years, and the Y-axis shows the number of articles published in a particular year.

Moreover, we further analyzed the current literatures to check whether they reflected/satisfied the two aspects of the ISA definition 1) Knowledge and Awareness and 2) *Activities and Compliance.* Our findings show that majority (n = 18) of the studies considered the first aspect, while only (n = 6) of the works focused on the second aspect. (Activities and Compliance). Out of all, few works (n = 5) take into account both the aspects of ISA definition. Regarding the sample size, we only reported the number of participants recruited for the main survey. In addition, results show that majority of the articles (n = 15) were conducted in universities/colleges, and the participants were students, managers, academicians, and administrative staff. Six of the studies were conducted in private and public organizations, and the participants were employees, executive managers, and administrative staff. Thus, we can conclude that researchers considered two contexts (academia and industry) while developing or validating their scales. Two of the articles [68,71] did not mention any particular context and considered general internet or social media users as their participants. Another aspect we investigated is the geographical distribution of the articles. Since ISA is related to human factors that are characterized by subjectivity as well as cultural variations, it will be interesting to observe that current ISA scales originate from which geographical regions. The findings indicate that studies come from five continents across the globe. For example, eight studies were conducted in Europe (Turkey = 5, Croatia = 1, Sweden = 1, and Norway = 1). Three studies came from Australia alone, three from Asia (Malaysia = 1, Indonesia = 1, and China (Hong Kong = 1)), and four were conducted in the North America (USA = 3 and Canada = 1). Lastly, three articles came from the African continent (South Africa = 3), and three studies were conducted internationally (mixed countries). The country-based distribution of the articles is shown in Fig. 3, which indicates the worldwide interest in measuring the ISA concept. In addition, the year-based distribution of the selected articles is also depicted in Fig. 4. Around 58% of the articles (n = 14) were published in the past seven years, demonstrating that measuring ISA is still quite a young research field, and more efforts are needed in this aspect.

### 4.2 RQ_2_. What are the dimensions and sub-dimensions of ISA that researchers considered while developing their scales?

**Fig. 5:**
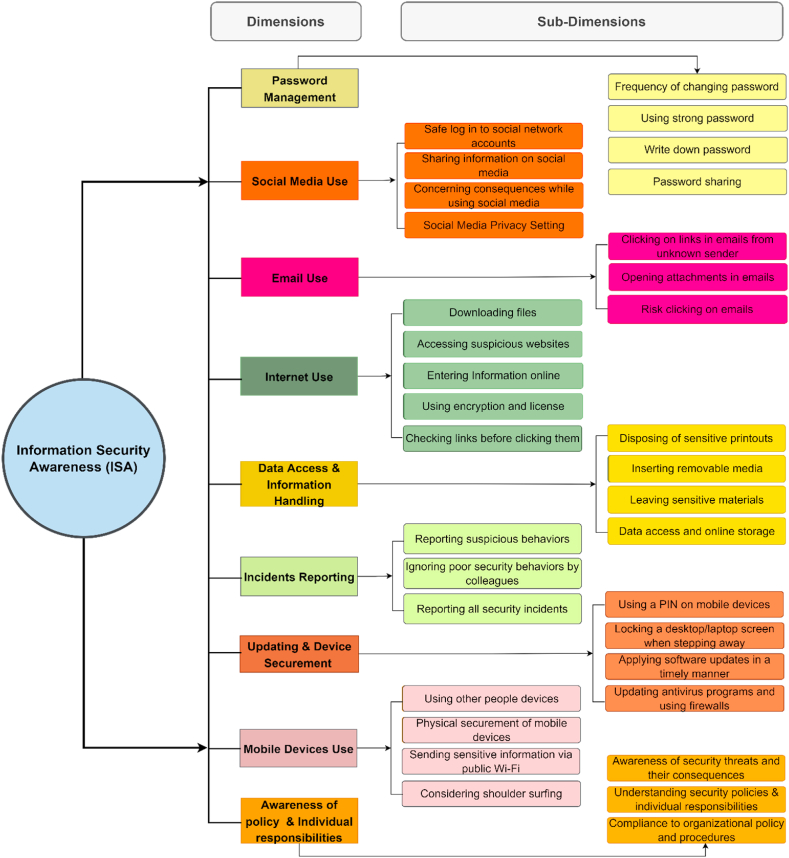
Dimensions of the ISA and their respective Sub-dimensions, based on the selected 24 articles.

We strongly feel that it is important to identify all the dimensions that current research has considered while developing scales on ISA. By analyzing all the selected articles, a total of nine dimensions are identified: password management, social media use, email use, internet use, data access and information handling, incident reporting, updating and device securement, and awareness of policies and individual responsibilities. Further, in order to understand the conceptual underpinning of each dimension, we tried to assign sub-dimensions to each one of them. As a result, a total of 34 sub-dimensions are identified. All the dimensions and their sub-dimensions are depicted in Fig. 5. The identified dimensions are briefly described below.

1) **Password management** refers to the awareness, usage, and management of passwords in general. Some examples include how a computer user can create a good and strong password, change it regularly, not share the work password with others, and be aware of the negative consequences.
2) **Social Media Use** is mainly related to the awareness and usage of social networking sites (e.g., Facebook, Instagram, LinkedIn, etc.). For instance, not accessing these sites during work time in organizations, considering the negative consequences before posting private and sensitive information, and regularly updating the privacy setting are some of the aspects considered by this dimension.
3) **Email Use** is relevant to the awareness and usage of emails. For example, employees in organizations should be aware not to click on links in emails from an unknown sender and consider not to download risky attachments (files) into a work computer. Further, social engineering is one of the most dangerous attacks, which is mainly carried out by phishing emails, so employees should understand not to be deceived by hackers.
4) **Internet Use** refers to the awareness of internet usage and accessing suspicious websites in general (downloading safe files, not accessing any suspicious websites, or not entering private and sensitive information online). Furthermore, internet users should understand the safe sources from which they download any file, not give private information on any website, and use content filtering programs.
5) **Data Access and Information Handling** refer to how a computer user can handle sensitive information and access or store data using online storage. For example, awareness of leaving sensitive materials (e.g., documents), downloading files from sources without checking their authenticity, and shredding sensitive printouts.
6) **Incident Reporting** is another dimension of information security awareness. It generally refers to reporting any security incident happening in a particular organization. Some examples include reporting suspicious behaviors of someone in the workplace, reporting the violation of the security rules of co-workers, or experiencing any security data breaches or incidents that should be reported.
7) **Device Securement and Updating** is a dimension where users/employees should understand and be aware of devices' security and regularly update the required software. For instance, internet users should regularly update the necessary software like antivirus, set a computer screen or other mobile devices to automatically lock while not using them, and use a password/passcode to unlock a computer or other mobile devices.
8) **Mobile Device Use** refers to the awareness of proper usage of mobile devices and keeping them secure [75]. For example, using secure networks while sending important emails, considering shoulder surfing while working on a sensitive document, physical securement of mobile devices like not leaving a work laptop unattended, etc.
9) **Awareness of policies and individual Responsibilities** is another dimension where employees in the organization should be aware and understand their responsibilities and follow all the organizations' security policies, rules, and procedures. For instance, awareness of the potential security threats, their negative consequences, and adherence to the organization's security rules and regulations.

**Fig. 6:**
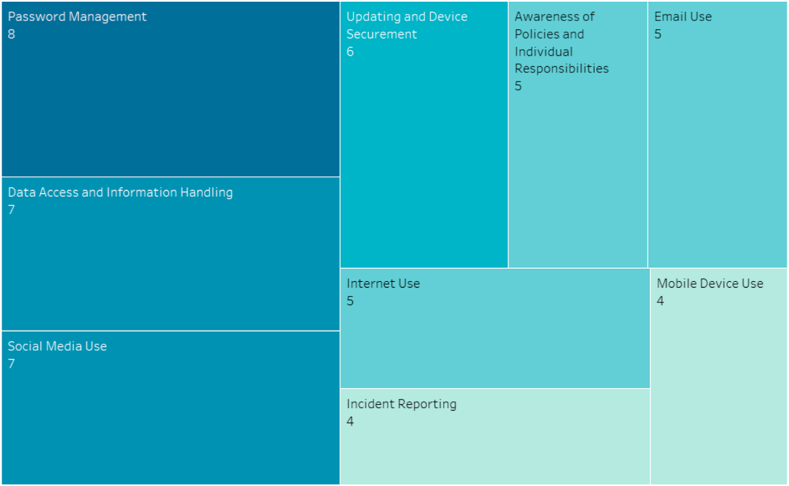
Frequency distribution of all the identified dimensions. The numbers in bracket (rectangle) indicate the frequency of their respective dimensions.

Furthermore, we were interested to check how frequently the identified nine dimensions shown above were used in current research. The findings show that not all the dimensions have been used equally by researchers while developing their respective scales. For instance, the dimensions of incident reporting and mobile device use are employed less frequently, whereas password management has the highest frequency of occurrence. The frequency distribution of the reported dimensions is shown in Fig. 6 in the form of a structure chart. Besides, we identified the publication venues, publication types, publishers, together with citations of all the 24 articles, which is presented in Table 4. That will help cybersecurity researchers to know where the leading authors on this topic have published.

**Table 4:** Publication venue, publication type, publisher, and citation details of the 24 articles.

Publication Venue	Publication Type	No of Articles	Publisher	Collective Citations*
Computers & Security	Journal	5	Elsevier	1722
Decision Support Systems	Journal	2	Elsevier	124
MIS Quarterly	Journal	1	Management Information Systems Research Center	2525
Annual ACM Conference on Human Factors in Computing Systems	Conference	1	ACM	298
Information Management and Computer Security	Journal	1	Emerald Insight	143
Pacific Asia Conference on Information Systems	Conference	1	Association for Information Systems (AIS)	60
Information Security Journal a Global Perspective	Journal	1	Taylor & Francis	47
International Convention on Information Communication and Electronic Technology	Conference	1	IEEE	33
Issues in Information Systems	Journal	1	International Association for Computer Information Systems	20
Library Collections Acquisition and Technical Services	Journal	1	Elsevier	14
Jurnal SISFOKOM	Journal	1	Lembaga Penelitian dan Pengabdian Masyarakat ISB Atma Luhur	13
Information Development	Journal	1	SAGE	7
Safety and Reliability–Safe Societies in a Changing World	Journal	1	Taylor & Francis	6
International Journal of Mechanical Engineering and Technology	Journal	1	IAEME	6
International Symposium on Human Aspects of Information Security & Assurance	Conference	1	Springer	5
Online Information Review	Journal	1	Emerald Insight	5
International Conference on Cyber Situational Awareness Data Analytics and Assessment	Conference	1	IEEE	4
Athens Journal of Mass Media and Communications	Journal	1	Academic Journals	0
The Journal of Academic Social Science Studies	Journal	1	Macrothink Institute	0

**Note:** * = Citation as per Google Scholar dated 27/01/2023.

### 4.3 RQ_3_. What is the methodological thoroughness/rigor of the scale development procedures of the measurement scales?

To answer the third research question, all the 24 selected articles are evaluated based on the first two phases (item generation and scale development) of the evaluation framework. The two phases consist of 15 criteria utilized for assessing the methodological thoroughness/rigor of the existing scale development process. The results are outlined in Table 5. It is very alarming to see that in a critical scenario like cybersecurity and ISA more than 50% of the articles could not fulfill even 50% of the criteria. In terms of overall fulfilment of each criterion, the major drawback lies in the scale development phase (phase 2). For example, only 8% of the studies have presented details about item retention or deletion and optimizing the scale length. Likewise, a meagre 21% of the articles perform the act of data cleaning or report the various distribution statigstics and issues related to bias and multicollinearity. Only 18% of the articles verify whether the data they collect is suitable for doing factor analysis or not. Likewise, only 33% of the articles do a pilot testing, which is important for having an initial idea about the item distributions and re-modify some items before conducting the actual survey. Another issue lies with the sampling frame selected for doing CFA, where only 37% of the articles fulfill the selected criterion. An overwhelming 63% of the articles either do not carry out a CFA or do it on the same sample as that of EFA, which should be strictly avoided.Most of the strength area of the existing scales belong to phase 1 (item generation) with regards to the conceptual definition and dimension/sub-dimension identification. However, it should be noted that around 40% of the articles do not follow the dual inductive-deductive approach for item generation that is expected from good scales. Overall, the results depicted in Table 3 is a clear indication that there is room for several improvements as there are many grey areas of the current scales that have been developed for measuring ISA.

**Table 5:** Results based on the criteria relevant to the methodological rigor of the scale development procedures.

Ref#	Conceptual definition	Dimensions	Item Generation	Pre-Test Evaluation	Pilot Testing	Sample size	Data Cleaning	Factorability Tests	Factor Extraction	Factor Rotation	Factor Retention	Items Retention or Deletion	Optimizing the scale length	Sampling Frame for CFA	Model Fit indices	Total Fulfilled Criteria in %
[34]	1	1	0.5	0	0	0	1	0	0	0	0	0	0	0	0	23%
[35]	1	0.5	0.5	0	0	0.5	1	0	0.5	0	0.5	0	0	0	0	30%
[36]	1	1	0.5	0.5	0	1	0	0.5	0	0.5	0.5	0.5	0	1	1	53%
[54]	1	0	1	1	1	1	0	0	1	1	0	0	0	1	1	60%
[55]	1	1	0.5	0.5	0	1	0	0	0	1	0.5	0	0	0.5	1	47%
[56]	1	0.5	0.5	0.5	0	1	0	0.5	0	0	0	0	0	0	0	27%
[57]	1	1	1	1	1	1	0	0	1	1	0	0	0	0.5	0	57%
[58]	1	1	1	1	1	1	0.5	0	0.5	1	0	0	0	0	0	53%
[59]	1	1	1	1	1	1	0.5	0.5	0.5	0	1	0	0	0	0	57%
[60]	1	0.5	0.5	0.5	0	0	1	0	0	0	0	0	0	0	0	23%
[61]	0.5	1	0.5	1	0.5	0.5	0	0.5	1	1	1	0	0	0.5	1	60%
[62]	1	0.5	0.5	1	1	1	0	0	1	1	1	0	0	1	1	67%
[63]	0	0.5	0.5	0	0	0.5	0	0.5	0.5	0.5	0.5	0.5	1	0	0	33%
[64]	1	1	0.5	0.5	0	1	0	0	0.5	1	0.5	0.5	0	1	1	57%
[65]	1	1	0.5	0.5	1	1	0	0	0.5	0	0.5	0	0	0	0	40%
[66]	1	1	0.5	0.5	0	1	0	0	0.5	0.5	1	0.5	1	0.5	0	53%
[67]	1	1	0.5	1	0.5	1	0	0	0.5	0	0.5	0	0	0.5	0	43%
[68]	1	1	0.5	1	0	1	0	0	0	0	0.5	0	0	0.5	1	43%
[69]	1	1	1	1	0	1	0	0.5	0.5	1	1	0	0	0.5	1	63%
[70]	1	1	0.5	0.5	0.5	0	0	0.5	1	0	1	0	0	0	0	40%
[71]	1	1	0.5	0	0	0.5	1	0	0	0	0	0	0	0	0	26%
[72]	1	1	0.5	0.5	0.5	1	0	0.5	0.5	0.5	0.5	0	0	1	1	57%
[73]	1	1	0.5	0.5	0	1	0	0.5	0.5	0	0.5	0	0	0.5	1	47%
[74]	1	0.5	0.5	0	0	0.5	0	0	0	0.5	0.5	0	0	0	0	23%
Total	93%	85%	60%	58%	33%	77%	21%	18%	43%	43%	48%	8%	8%	37%	42%	–

### 4.4 RQ4. What is the reported reliability and validity of the identified scales?

With respect to the reliability and validity of the scales, the results are presented in Table 6. As mentioned before in our evaluation framework, four measures are considered for this purpose: internal consistency (α value), convergent validity, discriminant validity, and criterion validity. None of the present scales fulfill 100% of the criteria, in fact, 75% is the maximum level reached. Surprisingly, roughly 71% (n = 17) of the articles fulfilled a maximum of 50% of the presented criteria. Internal consistency is the most reported reliability measure at 87%, while none of the scales report criterion validity. Convergent validity is also reported by only 37% of the existing scales.

**Table 6:** Results based on the criteria relevant to the quality of the measurement scales.

Ref#	Internal Consistency	Criterion Validity	Convergent Validity	Discriminant validity	Total Fulfilled Criteria in %
[34]	1	0	0	0	25%
[35]	1	0	0	1	50%
[36]	1	0	1	1	75%
[54]	1	0	1	1	75%
[55]	1	0	0	0	25%
[56]	1	0	0	0	25%
[57]	1	0	1	1	75%
[58]	1	0	0	1	50%
[59]	0.5	0	1	0	37%
[60]	1	0	0	0	25%
[61]	0.5	0	1	1	62%
[62]	1	0	1	1	75%
[63]	1	0	0	0	25%
[64]	1	0	0	1	50%
[65]	0.5	0	0	1	37%
[66]	1	0	1	1	75%
[67]	0.5	0	0	1	37%
[68]	1	0	1	1	75%
[69]	1	0	0	1	50%
[70]	1	0	0	0	25%
[71]	0	0	1	0	25%
[72]	1	0	0	0	25%
[73]	1	0	0	0	25%
[74]	1	0	0	0	25%
Total	87%	0%	37%	54%	–

As evident from the results overall, the implementation of validity analysis of the scales is underdeveloped, which makes it difficult to judge the appropriateness of the scales developed. An in-depth discussion about the results is presented in the next section.

## 5 Discussion

The current research aims to comprehensively review the existing measurement scales related to ISA. In this regard, 24 studies are identified. We initiated to investigate the current state-of-art of these scales together with the dimensions and sub-dimensions researchers considered while developing these. Moreover, we developed our own evaluation framework to evaluate the rigor of the existing scale development procedure together with the quality of the scales. The major findings of this study are discussed below, along with the recommendations that need to be considered by researchers in the future. We base the discussions on the three distinct phases of scale development that we presented previously in Fig. 2.

### 5.1 Item generation

Identifying and articulating the constructs with a clear conceptual definition is the first significant step in scale development. Approximately (93%) of the selected studies reported the conceptual definition of their constructs, which is a substantial initiation for scale development. This finding is consistent with current research as in Ref. [42], where majority of the articles reported the presence of all the essential definitions for their constructs. Although reporting the definitions is essential, the quality of these definitions is equally important. Moreover, before developing any new scale it must be ensured that there are no existing scales that will sufficiently use and serve the same objective [38]. If there is a similar scale available, authors need to justify why the development of a new scale is necessary and how it will differ from the existing scales.

One problem with the current ISA scales is that there are a very few of them which are novel, either in terms of their scope or the context in which they are being used. For example, only 2% of the potential articles related to ISA scales made it to our final corpus, since these articles simply re-used existing scales while measuring various forms of human behavior. Scale development is an iterative process, and it is an acknowledged fact that with multiple iterations the quality of the scales improves, either in terms of the scale length or their psychometric properties or even capturing some new dimensions. However, related to ISA we could not find any such efforts from the research community, and it seems that the attempts towards measuring various aspects of ISA are rather fragmented. Therefore, further research into this aspect should not only focus on new scales, but also modify and improve the quality of the existing ones.

Our findings also indicate the presence of two ontologically distinct approaches (unidimensional and multi-dimensional) for measuring ISA. Majority of the studies (85%) identified several dimensions and measured ISA as a multi-dimensional construct. This result is similar to current research in Refs. [37,42]. Only one article [54] treated ISA as a unidimensional construct, where it was measured directly without any dimensions. In this regard we would like to refer to the commonly accepted definition(s) of ISA that we had previously outlined in the Introduction section that clearly mentioned two significant aspects. The first aspect relates to how well the users in an organization comprehend the importance and significance of information security issues and threats. The second aspect focuses on how well the users follow the organizations' privacy and security rules and policies while performing various cyber activities. It becomes evident that there are at least two different aspects of ISA with one focusing on the awareness and knowledge aspect of the users, while the other one focusing on the activities performed and compliance of the users. Therefore, conceptually ISA has a multi-dimensional flavor that is evident from the results also. However, our findings also reveal that majority of the existing ISA scales (75%) focus on the first aspect of awareness and knowledge, while only 25% of the scales focus on the second aspect of activities performed and adherence to the rules/policies. Likewise, only 20% of the studies focus on both these aspects. Therefore, it becomes evident that the current ISA scales are not comprehensive enough, and they lack maturity since majority of them are not able to capture the actual usage behavior of the users and whether such behavior complies with the organizational policies. From a practical view-point administration of such ISA scales is of little significance, and future research must consider this aspect.

Furthermore, our analysis reveals the presence of nice unique dimensions (Fig. 5) that current ISA scales consider, however, not all the dimensions are given equal importance (Fig. 6). Some aspects of ISA like incident reporting and mobile device usage have been less investigated by the current scales. Smartphones are now ubiquitous, and they pose a serious challenge to any organization's security as typically these devices are not managed by the organizations. Smartphones and other handheld computing devices like tablets, laptops, etc. come under the Bring Your Own Device (BYOD) paradigm that is unavoidable in today's organizational environment. Although BYOD brings in new and unique challenges in terms of an organization's privacy and security, its coverage by current ISA scales is insufficient. Likewise, for any organization cybersecurity incident reporting is a part of their layered defense system providing a framework for effective incident reporting. Considering the growth of various types of phishing attacks in the recent years for example, incident reporting becomes very important. Therefore, cybersecurity researchers must be more proactive in including these under explored dimensions when assessing ISA.

In terms of item generation, our results showed that only 20% of the studies reported comprehensively utilizing both the inductive and deductive approaches. The remaining 80% of the studies employed only one of the above approaches. While there is no clear-cut rule stating the superiority of one of the approaches over another, yet it is advisable to use a mixed-method approach considering the importance of item generation in the scale development process and ensuring an in-depth coverage of the constructs being measured. Another limitation we observed was in terms of the ratio of the final scale size to the size of the initial item pool. Current literatures on scale development such as [21,41] recommended that the initial set of items should be at least 3 to 4 times the final expected scale length. However, about 30% of the ISA scales did not maintain this recommended ratio. This might be a serious issue, especially in the item reduction phase to ensure the parsimonious nature of the scale.

### 5.2 Scale development

During the second phase of scale development our findings show that only 37.5% of the studies conducted pre-tests based on both expert feedback and the target population, while 42% of the studies focused on any one group. Ideally pre-tests involving experts are very important as they can give their opinion both from a methodological perspective and domain knowledge. On the other hand, the target population consists of the potential users of the scale. One peculiar thing that we observed in this case was most of the potential users were students in an academic scenario. However, considering only students in the pre-test phase is not a good idea, because they might not be qualified enough to evaluate the scales. Besides checking the complicated wording of the items, their representativeness, vagueness, presence of biased questions, and overall technical quality there are some other basic conditions that must be checked to ensure content validity. For instance, it is very important to check the content adequacy and whether the scales can reflect the construct and measure what they were supposed to measure [76], which is difficult for the student population to evaluate because of their inexperience. Hence, it is strongly recommended that future security research should consider qualified expert judges (who have sufficient domain knowledge and a great experience in scale development) [24,40]. Some qualitative methods, such as interviews, focus groups, and group discussions, can play a significant role in this case. Our investigation also demonstrated that a few studies (20.5%) did not do pre-test evaluation. This is unfortunate since pre-test evaluation is an essential aspect of the scale development process that needs to be conducted before the launch of the main survey.

For this phase, our findings revealed that majority of the works (67%) did not conduct a pilot test. Only 25% of the studies conducted a pilot test and presented the EFA results too, while remaining 8% of the studies did not report the EFA results. However, this is not a good practice, since EFA allows to determine how data will fall around each factor, identify what items should be deleted, and recognize if any items are missed. It further assists researchers in ensuring that items are meaningful to the target population before the actual survey is conducted [38]. Majority of the current ISA scales have missed these important issues that future scales should keep in mind.

Before conducting the main survey, deciding on sample size is imperative as a sufficient sample can help to minimize the measurement errors [77]. In this regard, our findings demonstrated that most of the studies (77%) maintained the minimum threshold sample size of greater than 300 or minimum ratio of 5–10 participants per item. These results are similar to current findings in Ref. [42]. Factor analysis (EFA and CFA) needs a big and suitable sample size; thus, inadequate sample sizes not only result in unstable factors but also reduce generalizability [21]. After the data collection, cleaning the data (checking for missing data, outliers, and multicollinearity issues) is essential before doing any analysis. Unfortunately, our results indicated that roughly 79% of the ISA researchers did not report the data cleaning process. This finding is in direct violation of the best practices of scale development. ISA researchers should report how missing data was handled and carry out an outlier analysis together with reporting the precautionary steps undertaken to prevent multicollinearity issues during data collection. Additionally, statistical measures like Variance Inflation Factor (VIF) values should also be reported.

Before doing factor analysis, verifying the factorability of the data is vital to check whether the factor analysis should be implemented on the collected data. In this case, the correlation matrix, KMO test, and Bartlett's test of sphericity need to be done and reported. Our investigation showed that most of the articles (82%) did not examine these tests before the factor analysis. A similar finding was reported by authors in Ref. [37], where researchers did not often report that they have investigated these statistics. As current research suffers from not providing evidence for factorability tests, thus it is strongly recommended that ISA researchers should conduct all three statistics mentioned above prior to factor analysis. At least two of them (the KMO test and Bartlett's test) are mandatory. Moving on, conducting EFA is another substantial activity in scale development. Based on our evaluation framework, the rigor of EFA is assessed by checking five critical aspects: factor extraction method, factor rotation, factor retention, item deletion or retention, and optimizing the scale length. The first criterion under the umbrella of EFA was to check whether the existing research used any factor extraction method/s. Our analysis indicates that less than half of the studies reported the factor extraction methods (20% reported FA and 23% reported PCA).

Factor rotation is another criterion that is essential to determine the scale's factors dimensions more clearly. Our findings in this regard showed that few works (43%) reported the factor rotation process, while majority (57%) did not report it. This is another aspect of the scale development procedure that, currently, ISA researchers missed to conduct. Furthermore, there are two types of rotation methods: orthogonal and oblique. Our analysis found that most of the studies used Varimax (a type of orthogonal rotation) method. Authors should understand that although orthogonal is a widely used and well-known rotation method, but it forces factors to not correlate. For example, a Varimax rotation is biased as it pushes high factor loadings higher and low factors lower because they are not allowed to correlate [37]. Similarly, in case of cross loading, the percentage of orthogonal rotation is more than the Promax rotation [78]. Hence, the current literature recommends utilizing Promax, which is an oblique rotation method, instead of Varimax, as it more accurately represents models [21,47]. Consequently, whatever extraction method is chosen, ISA researchers should provide sufficient rationale for choosing either of the two methods, and the rotation method should not be biased against finding a general factor and create more cross-loadings in the procedure that might be a trouble.

Factor retention is another method to determine latent factors that fit a set of items and is a significant part of factor analysis. Our results demonstrate that less than half (48%) of studies reported factor retention while developing their scales. It must be clear that distinct criteria may be utilized for retaining the ideal number of factors based on the item loadings. In this regard, our analysis found that ISA researchers considered mostly the rule (eigenvalue >1) followed by the scree plot while identifying the optimal factors. Although both of the methods/rules are popular among the researchers, some studies, like [37,79], preferred the scree plot over the rule of eigenvalue and claimed that it is more accurate. Sadly, the rest (52%) of the articles in our review did not report factor retention. These findings are almost similar to the results reported by studies in Refs. [38,42], which stated that several articles that developed scales either missed doing this step or did not report it. Future research should take this into account to report all the methods mentioned above for retaining the ideal number of factors.

Retaining and deleting items that load onto one or multiple factors is also an essential aspect to be taken into account. Unfortunately, our findings revealed that only (8%) of the ISA studies reported the final factor structure. It is one of the main drawbacks and does not seem to be a good practice that security researchers missed conducting or reporting details about this step, although it is paramount. The way how to proceed with item deletion or retention is not complicated. If an item cross-loads onto multiple factors, such cross-loadings should have less than 0.15 difference from an item's highest factor loading [21,80]. Similarly, items should also be deleted if they load with the same minimum threshold values (or greater) across multiple factors. Although many scale development articles miss discussing or explicitly report details about this particular aspect, to ensure the reproducibility of research and acknowledge the fact that scale development is often an iterative process, publishing the final factor structure becomes extremely necessary as it offers different insights. Hence, this criterion should be considered in the future.

As a final step of the EFA, to ensure a good quality scale, it was also necessary to assess the trade-off between the length of the scale and its reliability. In this regard, our analysis indicated that very few (8%) ISA researchers considered this criterion while developing their scales. This is another shortcoming of the existing scales used to measure ISA. We would like to give some details regarding the importance, how to proceed with it, and reporting this specific criterion. Although longer scales are typically more reliable, it might be challenging to actually administer these scales commercially due to a lack of respondent motivation, time, and exhaustion [81]. Consequently, it is a reasonable idea to go for a trade-off by sacrificing a small degree of internal consistency to shorten the scale. For instance, if a factor has more than the expected number of items, then the researchers can delete the item that has the minimum loading or the item that has the least contribution to the internal consistency of the scale. Nevertheless, it must be mentioned that such optimizations should not degrade the quality of the factor structure, item communalities, or cross-loadings.

Once the factor structure is established via EFA representing the measurement model, a CFA should typically be conducted to confirm the hypothesized model. The thoroughness of CFA was evaluated based on sampling frame and model-fit indices. The sampling frame is one of the crucial criteria in scale development that dictates the quality of CFA. Our findings demonstrated that less than half (37%) of the ISA researchers took this into account to utilize two different data sets for EFA and CFA. Using the same date set for both EFA and CFA is one of the common mistakes. The comprehensive scale development studies insist that the same dataset should not be used for carrying out CFA that was used for conducting the EFA [21,50]. However, ISA researchers can keep in mind that based on the initial sample size or the number of items, different split ratios can be used that do not affect the quality of the data analysis. Otherwise, it is always recommended to carry out the CFA on a new sampling frame. Moreover, another significant issue is that all the requirements that we mentioned previously (evaluation framework) for selecting a suitable sample should still hold true for CFA. After the sampling frame, the next step was to check for the different fit-indices. In this regard, our findings revealed that more than half of the researchers (58%) did not report that they examined the fit indices, which are essential. This is strange as a huge percentage of scale developers in the domain of ISA missed to conduct or report the various fit indices. It is strongly recommended that besides reporting the chi-square test statistic and the associated degrees of freedom, researchers should also report the incremental, absolute, and predictive fit indices, which are presented in the evaluation framework.

### 5.3 Scale validation/evaluation

Reliability is one of the significant aspects of the scale development process, which is checked mainly by internal consistency. Fortunately, the findings of our analysis indicated that most (87%) of ISA researchers reported the internal consistency of their scales. Out of that, 18 studies considered both the Cronbach alpha test and Composite reliability (CR). The other five studies considered one of the methods only. Nevertheless, both these measures were selected as indicators of internal consistency because the way these are assessed is fundamentally different. For example, Cronbach's alpha considers all the factor loadings to be the same for all the items; however, CR takes into consideration the variable item factor loadings. Moreover, scholars should know that although Cronbach alpha is the most popular and accepted statistic for checking reliability, followed by CR, there are some other reliability statistics such as Raykov's rho, Revelle's, ordinal alpha, and beta that are under discussion among the researchers [46]. However, there was only one study in our review that did not report the internal consistency. Conducting and reporting reliability is strongly recommended by the existing literature [49,78]. Thus, researchers in the future must report all the necessary/recommended tests to ensure that the scales are reliable enough to be used.

Moreover, based on our evaluation framework, the validity is commonly checked by construct validity (criterion validity, convergent validity, and the discriminant validity). It is very unfortunate that none of the studies in our review investigated the criterion validity. This finding is consistent with the previous literature review [31], which stated, “although criterion validity is one of the strongest pieces of evidence for construct validity, it is usually the most difficult to obtain”. Moreover, the domain to measure ISA seems to be relatively young, which may be one of the reasons why ISA researchers did not conduct the criterion validity. In terms of the convergent validity, only a few (37%) of the studies conducted it, while the majority of them (53%) did not report. Convergent validity is a significant measure of validity, and it is the degree to which a construct measured by several methods achieves similar results. Researchers can calculate this validity through the factor weights (loadings) or an additional approach, the average variance extracted (AVE) coefficient [39]. Finally, the test of discriminant validity is only done by (54%) of the articles, while the minority of the articles did not conduct or report assessing the validity of their scales.

These findings revealed very weak evidence for the validity of the scales. One of the reasons why there is a lack of evidence for validation of ISA scales may be that the domain is relatively young, and the interest in the concept of ISA has significantly increased very recently. Researchers should keep in mind that scale validation is one of the essential factors in the information security domain. For instance, inadequately validated measurement scales may lead organizations to incorrectly assess users’ ISA [18]. Hence, decisions based on this may have devastating outcomes. Therefore, it is paramount that future research must focus more on the validity aspect of their scales by providing sufficient evidence to ensure that the scales are valid. In addition, we further checked whether researchers provided evidence for validity by conducting some experimental studies after developing their scale or if they only did it based on the statistical measure of data they collected. We found only one article [59] that further validated their scale by conducting an empirical phishing email study.

### 5.4 Observations and recommendations for future research

We already discussed the findings in the previous sections in details. Some important details regarding what we observed in the current ISA literature and recommendations for future research will be provided in this section. It was observed that many researchers did not provide proper justifications that why the development of new scales is necessary. Hence, it is significant in the future that ISA scale developers should ensure that there is no existing scales available that is used for the same objective, or they need to justify why the development of new scales is required. In addition, the focus of scale developers should not only be on the new scales, but rather they should try to modify and improve the quality of the existing scales by doing multiple iterations. Likewise, we also observed that current ISA-specific scales rarely covered both the aspects of ISA definition (a. Awareness and Knowledge, and b. Activities and Compliance), and there were few existing ISA-specific scales that included all the dimensions of ISA. Therefore, security researchers should consider all these aspects while developing scales in the future.

Furthermore, many security researchers failed to concentrate on both experts and potential users while conducting the pre-test evaluation. A strange thing was observed in this respect that most of the potential users were learners from universities/colleges. However, considering only learners is not a good idea because they might not be in a position (qualified enough) to evaluate the scales. Thus, future research should consider both qualified experts (well-educated and experienced people who can give their perceptions from theoretical, methodological, and practical perspectives, and have sufficient domain knowledge) and their potential users while conducting the Pre-test Evaluation. Another observation was that majority of the current ISA research either did not conduct or missed reporting data cleaning process and providing evidence for the factorability test, which is paramount while developing scales. Similarly, ISA scale developers considered only one dataset for carrying out EFA and conducting the CFA, which is a clear violation of scale development best practices. Security researchers must take into account all these significant points during the scale development procedure.

In addition, we observed that the existing ISA-specific scales showed fragile evidence for validity. For instance, it is strange that none of the articles examined the criterion validity. Similarly, less than half of the current studies missed to conduct or report the discriminant validity. Therefore, ISA scale developers must concentrate on these substantial aspects while validating their scales in the future. Also, it is recommended that security researchers should provide additional evidence for validity by conducting some experimental studies after developing their scales.

However, some significant points regarding the threat to validity are also observed, as some threats are associated with the design of the experiment, and others with social elements. *1) Design threats to construct validity* “covers issues that are related to the design of the experiment and its ability to reflect the construct to be studied" [82]. For example, the “inadequate preoperational explication of constructs”, which focuses on the constructs or dimensions that are not adequately defined before they are translated into measures. In this case, majority of the studies in our review considered this particular aspect, as it presented in the result section. *2) Social threats to construct validity*. “Such threats are concerned with issues related to the behavior of the subjects and the experimenters” [82]. For instance, the “Experimenter Expectancies”. Researchers can bias the outcomes of a study both intentionally and unintentionally based on what they expect from the study. This threat can be decreased by engaging various people who have no or different expectations to the experiment.

**Fig. 7:**
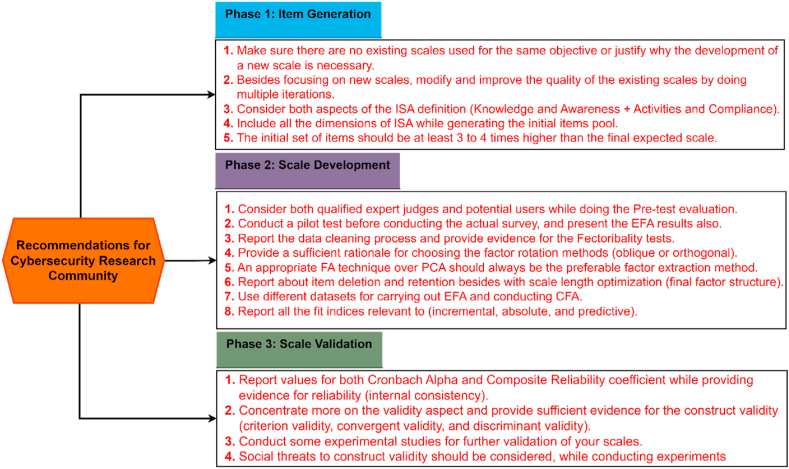
Recommendations to the cybersecurity research community.

For example, considering different people during the pre-test evaluation and the main survey, particularly while conducting EFA and CFA (considering two datasets with different sample population). In this respect, we found very few evidence that the current studies in our review take into consideration such social/subjective bias. This social/subjective bias can not only result from the psychology of the human beings, but also from the cultural differences of human beings. There are very few cross-cultural studies, which have done the scale development in a broader global perspective. This might be one of the ways through which we can remove the social/subjective bias, and therefore, security researchers should focus on these aspects in the future. Based on the discussion (sub-sections 1, 2, 3, 4, 5.4), we summarized the recommendations for future research in Fig. 7.

## 6 Conclusion

Scales manifest latent constructs and can measure attitudes, behaviors, and hypothetical scenarios we desire to exist due to our theoretical understanding of the world. Still, these factors/elements are difficult to be directly evaluated. Also, scales commonly capture behavior, a feeling, or an action that cannot be captured in a single variable or item; thus, many scales have been developed. Besides the availability of a huge number of scales, several incomplete scales in terms of (scale development and its validation process) are used to measure attitude, awareness, and behavioral attributes that are essential to our scientific investigation. With respect to the measurement scales of ISA, although there were few literature reviews that have focused on this aspect; however, they were limited and not comprehensive enough in terms of the methodological rigor of the scales, the initial conceptualization of them, data collection, and analysis during the development of the scales. Therefore, we provided a more holistic and systematic review of the existing literature that developed scales for measuring information security awareness and behavior. More objectively, four research questions were proposed, and a detailed methodology was presented.

By answering the first research question, 24 relevant articles were identified, and their basic information, like objectives, the target populations, their context, the initial items pool, and the proposed items, are all presented. Another aspect we investigated was the geographical distribution of the articles, where studies come from five continents across the globe, which shows the worldwide interest in measuring the concept of ISA. Regarding the second research question, a total of nine dimensions of ISA and 34 sub-dimensions are identified, which researchers considered while developing their scales. Moreover, we further found that not all the dimensions have been used equally by the researchers while developing their respective scales. For instance, dimensions of incidence reporting and mobile device use are employed less frequently, whereas password management has the highest frequency of occurrence.

For answering the third and fourth research questions, we proposed a comprehensive evaluation framework based on the core concepts presented by relevant literature. The framework has three phases and a total of 19 criteria. The first two phases of the framework consist of 15 criteria were used for answering the third research question. By analyzing all the selected articles, we found that apart from one study, all the rest presented the conceptual definition of their parent constructs at the beginning, which is a critical initiating point for establishing the items. Similarly, the majority of the works measured ISA as a multi-dimensional construct, while only one of the works measured it as unidimensional. It was also observed that ISA researchers rarely conducted both quantitative (pilot testing) and qualitative (pre-text evaluation) techniques while validating and refining the initial scales. Regarding the sample size, the majority of the studies used a sufficient sample size (abide by the rule of thumb). In this regard, we observed that ISA researchers considered participants from two major contexts: academia and industry while developing or validating their scales. Internet users from other important contexts like (home users) were missed. For selecting a more representative sample, ISA researchers should consider participants from all the three possible contexts. However, more than 78% of the works did not report both the data cleaning process and factorability tests. Additionally, several articles did not report some of the essential elements used for checking the rigor of EFA and CFA.

Consequently, in terms of the methodological thoroughness/rigor of the scale development procedures, none of the articles fulfilled all the criteria of our evaluation framework. Similarly, the analysis of the fourth research question showed that none of the studies used in this review fulfilled all the criteria regarding the validity of the evaluation framework. Although the reported reliabilities of the identified scales were good, as 87% reported the internal consistency of their scales, evidence for validities of the identified scales was very poor. Particularly none of the studies tested and reported the criterion validity. Therefore, scales for measuring ISA must be improved both in terms of the methodological thoroughness/rigor of the scale development procedures and the quality of the scales (reliability and validities).

Eventually, it is paramount for every organization to promote knowledge and awareness among their users by considering all the dimensions of ISA we have discussed, including vulnerabilities and attacks. Quality measurement scales can play a tremendous role in this case. Firstly, organizations can measure the ISA of their users in order to discover strengths and weaknesses; secondly, they can use this information to update their policies and provide suitable security awareness training programs for increasing the ISA level of their target users. Now, it is important to know what would be the best form (method) for information security awareness education. It depends on the specific target audience and goals of the program. However, according to these studies [9,15,83], some commonly used and effective forms are:

1. Online training modules: Convenient and accessible for a wide audience, easily customizable.
2. In-person workshops and presentations: Good for hands-on training and interaction with trainers and peers.
3. Gamification: Engaging and memorable, can increase participation and understanding of complex concepts.
4. Simulation exercises: Provides realistic scenarios for participants to apply their learning.
5. Brochures/posters, newsletters, and emails: Good for reminding things time to time.
6. Short videos and animations: Quick and visually engaging, can convey information effectively and simply.

Ultimately, a combination of different forms may be most effective in creating a comprehensive and effective information security awareness program. In this regard, some researchers believe that passive awareness forms, like oral presentations, emails, SMS messages, and newsletters, are insufficient for educating internet users [83,84]. Thus, there is a need to integrate more proactive forms (methods), such as, in-person training and workshops, simulations, and interviews, that are more effective and highly recommended.

## 7 Study limitations

Although we provided a comprehensive review of the existing literature related to measurement scales of ISA, still, our study itself is subject to some limitations that need to be considered. *First*, besides using the keyword combinations (search query) to capture both generic as well as specific research items for selecting the articles, maybe we missed some important articles developed scales for ISA that did not use these terms or relevant wording in the title or abstract of the article, which may impact our results. *Second*, it is significant that the readers should know that the low evaluation scores of some scales do not necessarily mean that those scales are of low quality. This may be because the researchers did not report some measures of their scales, like psychometric characteristics or other basic elements of the scale development procedures. *Third*, the methodological guidelines concerning threshold values and some of the statistical parameters may not be globally accepted. Therefore, the values are always relatively arbitrary, although we attempted to utilize a common denominator among various works. Fourth, our assessment was restricted to elements associated with the rigor of validation, which can be measured and objectively coded. However, a significant aspect of validation related to the theoretical issues of internal and external validity is missed to be investigated. Similarly, with respect to the analysis of items and reliability, we did not take into account some of the basic theories, such as classical test theory (CTT) and item response theory (IRT), that support the scale development procedures [21]. Although investigating these theories is out of the scope of our research, information on the employability of either one or both of them could assist in a more in-depth understanding of their main drawbacks. *Finally*, it is also worth mentioning that in our assessment, we followed the recommended methodological standards that mainly come from measurement scales developed in psychology, communication, marketing, social sciences, and health domains. Currently, there is no agreement on the “gold standards” of measurements in this specific domain of ISA.

## Author contribution statement

Rohani Rohan, MSc; Debajyoti Pal, Ph.D: Conceived and designed the experiments; Performed the experiments; Analyzed and interpreted the data; Wrote the paper.

Jari Hautamäki: Performed the experiments; Analyzed and interpreted the data.

Suree Funilkul, Ph.D.; Wichian Chutimaskul, Ph.D: Contributed reagents, materials, analysis tools or data.

Himanshu Thapliyal, Ph.D.: Contributed reagents, materials, analysis tools or data; Wrote the paper.

## Funding statement

Rohani Rohan was supported by King Mongkut's University of Technology Thonburi [Petchra Pra Jom Klao Doctoral Scholarship Research Funding].

## Data availability statement

Data will be made available on request.

## Declaration of interest’s statement

The authors declare no competing interests.

## References

[1] Bukauskas L. (2023).

[2] Keshavarzi M., Ghaffary H.R. (2023). An ontology-driven framework for knowledge representation of digital extortion attacks. Comput. Hum. Behav..

[3] Solomon A., Michaelshvili M., Bitton R., Shapira B., Rokach L., Puzis R., Shabtai A. (2022). Contextual security awareness: a context-based approach for assessing the security awareness of users. Knowl. Base Syst..

[4] Alzubaidi A. (2021). Measuring the level of cyber-security awareness for cybercrime in Saudi Arabia. Heliyon.

[5] Shaikh F.A., Siponen M. (2023). Information security risk assessments following cybersecurity breaches: the mediating role of top management attention to cybersecurity. Comput. Secur..

[6] Hasan S., Ali M., Kurnia S., Thurasamy R. (2021). Journal of Information Security and Applications Evaluating the cyber security readiness of organizations and its influence on performance. J. Inf. Secur. Appl..

[7] Yeoh W., Wang S., Popovič A., Chowdhury N.H. (2022). A systematic synthesis of critical success factors for cybersecurity. Comput. Secur..

[8] Zwilling M., Klien G., Lesjak D., Wiechetek Ł., Cetin F. (2020). Cyber security awareness , knowledge and behavior : a comparative study cyber security awareness , knowledge and behavior : a comparative study. J. Comput. Inf. Syst..

[9] Katsikeas S., Johnson P., Ekstedt M., Lagerström R. (2021). Research communities in cyber security: a comprehensive literature review. Comput. Sci. Rev..

[10] Ayyoub H.Y., AlAhmad A.A., Al-Serhan A., Al-Abdallat M.F., Al-Muheisen E., Boshmaf H., Abu-Taleb Y.A., Alqudah Y.O., Alshamaileh Y. (2022). Awareness of electronic crimes related to E-learning among students at the University of Jordan. Heliyon.

[11] Hina S., Dominic P.D.D. (2020). Information security policies' compliance: a perspective for higher education institutions. J. Comput. Inf. Syst..

[12] Ogonji M.M., Okeyo G., Wafula J.M. (2020). A survey on privacy and security of Internet of Things. Comput. Sci. Rev..

[13] Rahman T., Rohan R., Pal D., Kanthamanon P. (2021). Human factors in cybersecurity: a scoping review. ACM Int. Conf. Proceeding Ser..

[14] Rohan R., Funilkul S., Pal D., Chutimaskul W. (2021). Understanding of human factors in cybersecurity : a systematic literature. Review.

[15] Gkioulos V., Chowdhury N. (2021). Cyber security training for critical infrastructure protection: a literature review. Comput. Sci. Rev..

[16] Chaudhary S., Schafeitel-Tähtinen T., Helenius M., Berki E. (2019). Usability, security and trust in password managers: a quest for user-centric properties and features. Comput. Sci. Rev..

[17] Da Veiga A., Martins N. (2015). Information security culture and information protection culture: a validated assessment instrument. Comput. Law Secur. Rep..

[18] Rahim N.H.A., Hamid S., Kiah L.M., Shamshirband S., Furnell S. (2015). A systematic review of approaches to assessing cybersecurity awareness. Kybernetes.

[19] Rohan R., Funilkul S., Pal D., Thapliyal H. (2021). Humans in the loop: cybersecurity aspects in the consumer IoT context. IEEE Consum. Electron. Mag..

[20] Pal D., Funilkul S., Papasratorn B. (2019). Antecedents of trust and the continuance intention in IoT-based smart products: the case of consumer wearables. IEEE Access.

[21] Morgado F.F.R., Meireles J.F.F., Neves C.M., Amaral A.C.S., Ferreira M.E.C. (2017). Scale development: ten main limitations and recommendations to improve future research practices. Psicol. Reflexão Crítica.

[22] Fertig T., Schütz A.E. (2020). About the measuring of information security awareness: a systematic literature review. Proc. Annu. Hawaii Int. Conf. Syst. Sci. 2020-Janua.

[23] Alotaibi M., Alfehaid W. (2018).

[24] Assenza G., Chittaro A., De Maggio M.C., Mastrapasqua M., Setola R. (2020). A review of methods for evaluating security awareness initiatives. Eur. J. Sci. Res..

[25] Liberati A., Altman D.G., Tetzlaff J., Mulrow C., Gøtzsche P.C., Ioannidis J.P.A., Clarke M., Devereaux P.J., Kleijnen J., Moher D. (2009).

[26] Long H.A., French D.P., Brooks J.M. (2020). Optimising the value of the critical appraisal skills programme (CASP) tool for quality appraisal in qualitative evidence synthesis. Res. Methods Med. Heal. Sci..

[27] Rohan R., Pal D., Funilkul S. (2020). Gamifying MOOC's a step in the right direction?: a systematic literature review. ACM Int. Conf. Proceeding Ser..

[28] Kitchenham B., Pretorius R., Budgen D., Brereton O.P., Turner M., Niazi M., Linkman S. (2010). Systematic literature reviews in software engineering-A tertiary study. Inf. Software Technol..

[29] Zhao M., Liu W., Naser A., Saif M., Wang B., Rupa R.A., Islam K.M.A., Rahman S.M.M., Hafiz N., Mostafa R. (2023).

[30] Pattinson M., Butavicius M., Parsons K., McCormac A., Calic D., Jerram C. (2016). The information security awareness of bank employees. Proc. 10th Int. Symp. Hum. Asp. Inf. Secur. Assur. HAISA.

[31] Hadlington L. (2017). Human factors in cybersecurity ; examining the link between [ 3 _ TD $ IF ] Internet addiction , impulsivity , attitudes towards cybersecurity , and risky cybersecurity behaviours. Heliyon.

[32] Yan Z., Robertson T., Yan R., Park S.Y., Bordoff S., Chen Q., Sprissler E. (2018). Finding the weakest links in the weakest link: how well do undergraduate students make cybersecurity judgment?. Comput. Hum. Behav..

[33] Calic D., Pattinson M., Parsons K., Butavicius M., McCormac A. (2016). Naïve and accidental behaviours that compromise information security: what the experts think. Proc. 10th Int. Symp. Hum. Asp. Inf. Secur. Assur. HAISA.

[34] Wijayanto H., Prabowo I.A. (2020). Cybersecurity vulnerability behavior scale in college during the covid-19 pandemic. J. Sisfokom (Sistem Inf. Dan Komputer)..

[35] Muhirwe J. (2016). Cybersecurity awareness and practice of next generation corporate technology users. Issues Inf. Syst..

[36] Arpaci I., Sevinc K. (2021). Development of the cybersecurity scale (CS-S): evidence of validity and reliability. Inf. Dev..

[37] Carpenter S. (2018). Ten steps in scale development and reporting: a guide for researchers. Commun. Methods Meas..

[38] Boateng G.O., Neilands T.B., Frongillo E.A., Melgar-Quiñonez H.R., Young S.L. (2018). Best practices for developing and validating scales for health, social, and behavioral research: a primer. Front. Public Health.

[39] Gilbert J., Churchill A. (2013). A paradigm for developing better measures of marketing constructs. J. Mar. Res..

[40] Hinkin T.R. (1995). A review of scale development practices in the study of organizations. J. Manag..

[41] C.T. DeVellis, R. F., & Thorpe, Scale Development: Theory and Applications, Fifth, SAGE, London, n.d.

[42] Orehek Š., Petri G. (2020).

[43] Pal D., Arpnikanondt C., Razzaque M.A., Funilkul S. (2020). To trust or not-trust: privacy issues with voice assistants. IT Prof.

[44] Henson R.K., Roberts J.K. (2006). Use of exploratory factor analysis in published research: common errors and some comment on improved practice. Educ. Psychol. Meas..

[45] Pal D., Arpnikanondt C., Razzaque M.A. (2020). Personal information disclosure via voice assistants: the personalization–privacy paradox. SN Comput. Sci..

[46] Costello A.B., Osborne J.W. (2005). Best practices in exploratory factor analysis: four recommendations for getting the most from your analysis. Practical Assess. Res. Eval..

[47] Hendrickson A.E., White P.O. (1964). Promax: a Quick method for rotation to oblique simple structure. Br. J. Stat. Psychol..

[48] Rohan R., Pal D., Funilkul S., Chutimaskul W., Eamsinvattana W. (2021). How gamification leads to continued usage of MOOCs? A theoretical perspective. IEEE Access.

[49] Dokument D., Nutzung D., Alexandre J.S., Arens A.Katrin, Marsh Herbert W., CON SINTAXIS Morin (2016).

[50] Steenkamp J.E.M., Maydeu‐Olivares A. (2022). Unrestricted factor analysis: a powerful alternative to confirmatory factor analysis. J. Acad. Market. Sci..

[51] King-Kallimanis B.L., Oort F.J., Nolte S., Schwartz C.E., Sprangers M.A.G. (2011). Using structural equation modeling to detect response shift in performance and health-related quality of life scores of multiple sclerosis patients. Qual. Life Res..

[52] Hu L.T., Bentler P.M. (1999). Cutoff criteria for fit indexes in covariance structure analysis: conventional criteria versus new alternatives. Struct. Equ. Model..

[53] Jackson D.L., Gillaspy J.A., Purc-Stephenson R. (2009). Reporting practices in confirmatory factor analysis: an overview and some recommendations. Psychol. Methods.

[54] Bulgurcu B., Cavusoglu H., Benbasat I. (2010). Information security policy compliance: an empirical study of rationality-based beliefs and information security awareness. MIS Q. Manag. Inf. Syst..

[55] Da Veiga A., Eloff J.H.P. (2010). A framework and assessment instrument for information security culture. Comput. Secur..

[56] Maidabino A.A., Zainab A.N. (2012). A holistic approach to collection security implementation in university libraries. Libr. Collect. Acquisit. Tech. Serv..

[57] Rocha Flores W., Ekstedt M. (2016). Shaping intention to resist social engineering through transformational leadership, information security culture and awareness. Comput. Secur..

[58] Parsons K., McCormac A., Butavicius M., Pattinson M., Jerram C. (2014). Determining employee awareness using the human aspects of information security questionnaire (HAIS-Q). Comput. Secur..

[59] Parsons K., Calic D., Pattinson M., Butavicius M., McCormac A., Zwaans T. (2017). The human aspects of information security questionnaire (HAIS-Q): two further validation studies. Comput. Secur..

[60] Kruger H., Drevin L., Steyn T. (2010). A vocabulary test to assess information security awareness. Inf. Manag. Comput. Secur..

[61] Alnatheer M., Chan T., Nelson K. (2012). Understanding and measuring information security culture. Proc. - Pacific Asia Conf. Inf. Syst. PACIS.

[62] Chu A.M.Y., Chau P.Y.K. (2014). Development and validation of instruments of information security deviant behavior. Decis. Support Syst..

[63] Velki T., Solic K., Ocevcic H. (2014). Development of users' information security awareness questionnaire (UISAQ) - ongoing work, 2014 37th. Int. Conv. Inf. Commun. Technol. Electron. Microelectron. MIPRO 2014 - Proc.

[64] Egelman S., Peer E. (2015). Scaling the security wall : developing a security behavior intentions scale (SeBIS). Conf. Hum. Factors Comput. Syst. - Proc..

[65] Öğütçü G., Testik Ö.M., Chouseinoglou O. (2015). Analysis of personal information security behavior and awareness. Comput. Secur..

[66] Masrek M.N., Harun Q.N., Zaini M.K. (2018). The development of an information security culture scale for the development of an information security culture scale for the. Int. J. Mech. Eng. Technol..

[67] Nævestad T.O., Frislid Meyer S., Hovland Honerud J. (2018). Organizational information security culture in critical infrastructure: developing and testing a scale and its relationships to other measures of information security. Saf. Reliab. - Safe Soc. a Chang. World - Proc. 28th Int. Eur. Saf. Reliab. Conf. ESREL.

[68] Vishwanath A., Neo L.S., Goh P., Lee S., Khader M., Ong G., Chin J. (2020). Cyber hygiene: the concept, its measure, and its initial tests, Decis. Support Syst.

[69] Erdoğdu F., Gökoğlu S., Kara M. (2021). What about users?”: development and validation of the mobile information security awareness scale (MISAS). Online Inf. Rev..

[70] Gangire Y., Da Veiga A., Herselman M. (2020). Information security behavior: development of a measurement instrument based on the self-determination theory. IFIP Adv. Inf. Commun. Technol..

[71] Schoenherr J.R., Thomson R. (2021). The cybersecurity (CSEC) questionnaire: individual differences in unintentional insider threat behaviours, 2021. Int. Conf. Cyber Situational Awareness, Data Anal. Assessment, CyberSA.

[72] Güldüren C. (2021). http://acikerisim.ufuk.edu.tr:8080/xmlui/handle/123456789/2430%0Ahttp://acikerisim.ufuk.edu.tr:8080/xmlui/bitstream/handle/123456789/2430/6-%29%20Information%20security%20awareness%20scale%20%28sas%29%20for%20university%20students.pdf?sequence=1&isAllowed=y.

[73] Tosun N., Gecer A. (2022). A development, validity and reliability of safe social networking scale. Athen. J. Mass Media Commun..

[74] Kim E.B. (2013). Information security awareness status of business college: undergraduate students. Inf. Secur. J..

[75] Almarhabi K., Bahaddad A., Mohammed Alghamdi A. (2023). Security management of BYOD and cloud environment in Saudi Arabia. Alex. Eng. J..

[76] Guion R.M. (1977). Content validity-the source of my discontent. Appl. Psychol. Meas..

[77] MacCallum R.C., Widaman K.F., Zhang S., Hong S. (1999). Sample size in factor analysis. Psychol. Methods.

[78] Norris M., Lecavalier L. (2010). Evaluating the use of exploratory factor analysis in developmental disability psychological research. J. Autism Dev. Disord..

[79] Goretzko D., Bühner M. (2022). Robustness of factor solutions in exploratory factor analysis. Behaviormetrika.

[80] Goretzko D. (2022). Factor retention in exploratory factor analysis with missing data. Educ. Psychol. Meas..

[81] Vucaj I. (2022). Development and initial validation of digital age teaching scale (DATS) to assess application of ISTE standards for educators in K–12 education classrooms. J. Res. Technol. Educ..

[82] (2015). Suparyanto Dan Rosad.

[83] Cheng E.C.K., Wang T. (2022). Institutional strategies for cybersecurity in higher education institutions. OR Inf..

[84] Alharbi T., Tassaddiq A. (2021). Assessment of cybersecurity awareness among students of majmaah university, big data cogn. Comput. Times.

